# The purplish bifurcate mussel *Mytilisepta virgata* gene expression atlas reveals a remarkable tissue functional specialization

**DOI:** 10.1186/s12864-017-4012-z

**Published:** 2017-08-08

**Authors:** Marco Gerdol, Yuki Fujii, Imtiaj Hasan, Toru Koike, Shunsuke Shimojo, Francesca Spazzali, Kaname Yamamoto, Yasuhiro Ozeki, Alberto Pallavicini, Hideaki Fujita

**Affiliations:** 1 Department of Life Sciences, University of Trieste, Via Giorgieri 5, 34126 Trieste, Italy; 2 Department of Pharmacy, Faculty of Pharmaceutical Science, Nagasaki International University, 2825-7 Huis Ten Bosch, Sasebo, Nagasaki, 859-3298 Japan; 3 Department of Life and Environmental System Science, Graduate School of NanoBio Sciences, Yokohama City University, 22-2 Seto, Kanazawa-ku, Yokohama, 236-0027 Japan; 4 Department of Biochemistry and Molecular Biology, Faculty of Science, University of Rajshahi, Rajshahi, 6205 Bangladesh

**Keywords:** Mussel, Transcriptome, RNA-seq, Lectins, Phylogenomics, Bivalve, Mollusk

## Abstract

**Background:**

*Mytilisepta virgata* is a marine mussel commonly found along the coasts of Japan. Although this species has been the subject of occasional studies concerning its ecological role, growth and reproduction, it has been so far almost completely neglected from a genetic and molecular point of view. In the present study we present a high quality de novo assembled transcriptome of the Japanese purplish mussel, which represents the first publicly available collection of expressed sequences for this species.

**Results:**

The assembled transcriptome comprises almost 50,000 contigs, with a N50 statistics of ~1 kilobase and a high estimated completeness based on the rate of BUSCOs identified, standing as one of the most exhaustive sequence resources available for mytiloid bivalves to date. Overall this data, accompanied by gene expression profiles from gills, digestive gland, mantle rim, foot and posterior adductor muscle, presents an accurate snapshot of the great functional specialization of these five tissues in adult mussels.

**Conclusions:**

We highlight that one of the most striking features of the *M. virgata* transcriptome is the high abundance and diversification of lectin-like transcripts, which pertain to different gene families and appear to be expressed in particular in the digestive gland and in the gills. Therefore, these two tissues might be selected as preferential targets for the isolation of molecules with interesting carbohydrate-binding properties.

In addition, by molecular phylogenomics, we provide solid evidence in support of the classification of *M. virgata* within the Brachidontinae subfamily. This result is in agreement with the previously proposed hypothesis that the morphological features traditionally used to group *Mytilisepta* spp. and *Septifer* spp. within the same clade are inappropriate due to homoplasy.

**Electronic supplementary material:**

The online version of this article (doi:10.1186/s12864-017-4012-z) contains supplementary material, which is available to authorized users.

## Background

The purplish bifurcate mussel *Mytilisepta virgata* (Wiegmann, 1837), also known as *Septifer virgatus*, is a small bivalve mollusk species commonly found in the middle/upper intertidal zone of moderately wave-exposed shores along the coasts of Japan, Taiwan, and South Eastern China [1], between −10 and +70 cm above the mean tide level [2]. *M. virgata* is usually found in dense mussel beds, whose lower limit of vertical distribution is often determined by the association with the lower-intertidal mussel *Hormomya mutabilis* [3]. This mussel species developed remarkable morphological adaptations to cope with a wave-exposed environment, including a ventrally flattened, particularly resistant shell and stronger byssal attachment to the substrate compared to other subtidal and lower-intertidal sympatric mussel species [3–5]. Furthermore, *M. virgata* appears to be much more resistant to high temperature stress and air exposure than *M. edulis*, another invasive mussel species which preferentially occupies the lower part of the intertidal zone in the same geographical region [2]. Mature individuals are usually 45 mm long and live for approximately 4–5 years, although exceptional cases of 65 mm long specimen surviving up to 12 years have been recorded. Sexual maturity is reached after 12 months and, although fertility is maintained throughout the entire year, spawning events follow a bimodal pattern (the first one occurring in February–March, the second one in September–December) [6].

From a taxonomical point of view, *M. virgata* has long been considered as a member of the *Septifer* genus (and therefore named *S. virgatus*) and placed within the subfamily Septiferinae [7]. However, this clade was later found to be polyphyletic and, based on the revised hierarchical classification of NCBI Taxonomy, *M. virgata*, along with and most of the other species previously paced within this subfamily, has been moved to Mytilinae (Rafinesque, 1815). However, the current taxonomical classification still does not appear to accurately reflect the evolutionary history of these mussels. Indeed, more than a decade ago, molecular studies based on Cytochrome c oxidase subunit I (COI) first pointed out that *M. virgata* and the morphologically similar *Septifer excisus* (Wiegmann, 1837) pertained to two different clades within the order Mytiloida [8]. This observation is strongly supported by a recent study by Trovant and colleagues, which reported that COI and 18S/28S–based phylogeny identified both *M. virgata* and *Mytilisepta bifurcata* (Conrad, 1837) as members of the same clade, together with *Perumytilus purpuratus* (Lamarck, 1819) and *Brachidontes rostratus* (Dunker, 1857) (both pertaining to the Brachidontinae family), but distantly related to *Septifer bilocularis* [9]. Based on these results, the authors further suggested that *Mytilisepta* should be retained as a separate genus within Brachidontinae and that the septum structure involved in the insertion of the adductor muscle and used for the current morphological classification of different species within the *Septifer* genus is the product of homoplasy.

Apart from its disputed taxonomical placement, very limited scientific attention has been so far focused on *M. virgata*, with only a handful of studies dealing with its morphological adaptations [5], embryonic development [10], reproductive cycle [6] and population dynamics [2]. To date, only 31 nucleotide and 11 amino acid sequences have been deposited in public repositories for this species (data retrieved from NCBI, June 2017). These include several partial sequences used for phylogenetic analyses [9, 11, 12], microsatellites [13], a complete mitochondrial sequence (KX094521.1) and a few unrelated sequences referring to still unpublished manuscripts, highlighting the still nearly non-existing molecular knowledge of *M. virgata*, in stark contrast with the extensive investigations carried out in *Mytilus* spp. since the early 2000’s [14–18]. The only reliable data available about the genome of *M. virgata* is an assessment of its size made by flow cytometry. The calculated genome c-value, 1.08, further confirmed by a very similar estimate for the closely related *Mytilisepta keenae* (1.06) [19], reveals that the purplish Japanese mussel genome is about 2/3 the size of those of *Mytilus* spp. and among the smallest known mytiloid genomes, but quite in line with the average genome size for all bivalves (Animal Genome Size Database, http://www.genomesize.com/).

Due the narrow scientific interest posed so far on mussel species other than *Mytilus* spp., deep sea vent mussels (*Bathymodiolus* spp.) and *Perna viridis*, *M. virgata* represents an interesting alternative subject for the study of the evolution of Mytiloida and for the investigation of some peculiar gene family expansion events which specifically occurred in this lineage [20].

The main aim of the present work was to provide the first curated and publicly accessible genomic resource for this species. The de novo assembled and functionally annotated transcriptome, together with gene expression data from five different adult tissues, will serve as a sequence and gene expression database for future genetic and molecular studies. The data we present will provide a substantial contribution in the improvement of scientific studies in this marine mussel. While discussing the potential function of tissue-specific genes, we put a particular emphasis on expanded families encoding lectin-like proteins involved in carbohydrate recognition, a class of molecules with a great biotechnological potential which could find a practical application in many areas of biomedical and biological research [21, 22].

## Methods

### Collection of samples

**Fig. 1:**
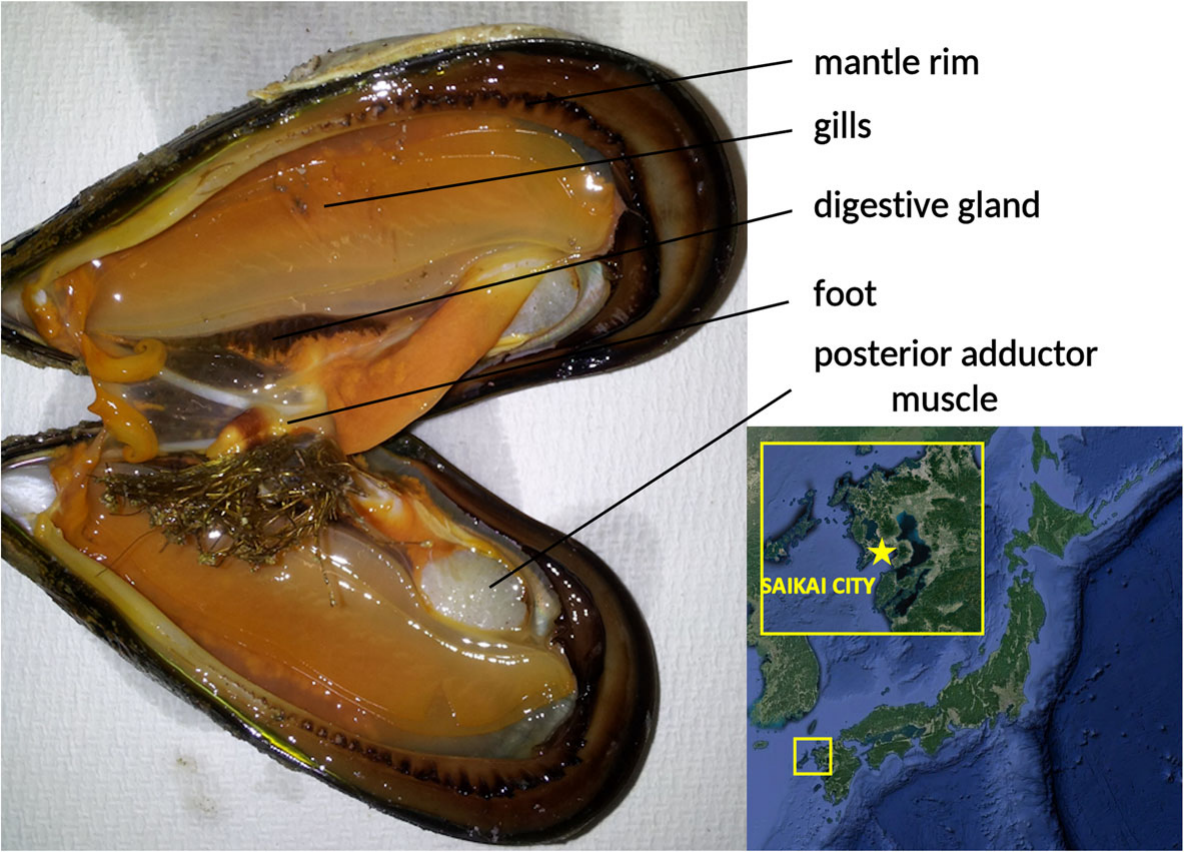
Internal anatomy of an adult *Mytilisepta virgata* specimen with indication of the five main tissues selected for this study and geographical location of the selected sampling site in Saikai city, Nagasaki prefecture (Japan)

The selected sampling site was a natural mussel bed found in a tide pool in Oshima, Saikai city (Nagasaki prefecture, Japan). Based on national and local fishing regulations, no permits were required for the collection of shellfish. Five adult mussels, whose shell size approximately ranged between 4 and 5 cm, were collected, dissected using razor blades and scissors, and tissues were immediately placed in RNAlater (Thermo Fisher Scientific, Waltham, USA). Namely, the following tissues were dissected and stored for subsequent RNA extraction: hemocytes (collected with a syringe), posterior adductor muscle, inner mantle, mantle rim, digestive gland, gills and foot (Fig. 1). The collected tissues were subsequently chopped into smaller parts weighting approximately 20 mg; for each of the sampled tissues, one of these slices of tissue was selected for each of the five specimens, placed in a vial containing TRIzol (Thermo Fisher Scientific, Waltham, USA) and homogenized. The total RNA, thereby representing a pool of five individual mussels, was extracted following the manufacturer’s instructions. The quality and quantity of the extracted RNAs were assessed with an Agilent 2100 Bioanalyzer (Agilent Technologies, Santa Clara, USA). Only samples whose RNA Integrity Number was > = 9 were selected for RNA sequencing. Unfortunately the quality of the RNA extracted from hemocytes and inner mantle was not sufficient to proceed with the preparation of Illumina sequencing libraries.

### Library preparation and sequencing of the samples

The preparation of cDNA libraries compatible with Illumina sequencing was carried out using the Lexogen SENSE mRNA-seq library prep kit v2 (Lexogen, Wien, Austria), aiming at an average fragment size of 400 nt. RNA-sequencing was performed at the DNA Sequencing Center of the Brigham Young University using a 2 × 125 bp paired-end strategy on a single lane of an Illumina HiSeq 2500 in high output mode with v4 reagents.

### De novo assembly, quality assessment and annotation

Raw reads were demultiplexed, imported in the CLC Genomics Workbench v.9.5 environment (Qiagen, Hilden, Germany) and trimmed according to base-calling quality scores and presence of residual sequencing adaptors. Resulting reads shorter than 75 bp were discarded. Trimmed reads were used as an input for Trinity v.2.3.2 [23] on the Galaxy platform [24] with default parameters. The minimum contig length was set at 200 bp. As suggested by previous studies [25, 26], in order to remove unreliable sequences likely originated from excessive fragmentation of longer mRNA molecules or residual contamination from exogenous RNA, contigs displaying a very low sequencing coverage were removed; this procedure was based on a threshold of TPM = 1, calculated with the back-mapping of all trimmed reads on the full assembled transcriptome using the software Kallisto v0.43.0 [27]. All the contigs that did not reach this threshold were discarded. Contigs originated from the assembly of mitochondrial and ribosomal RNAs were identified by BLASTn [28] using the sequences KX094521.1 (mitochondrial) and KJ453817.1 (ribosomal) as queries (e-value threshold 1E^−10^). The quality of the transcriptome was assessed with BUSCO v.2 [29] based on the set of conserved orthologous genes of the Metazoa lineage according to OrthoDB v.9 [30].

For annotation purposes, only the longest transcript per gene was selected and subjected to virtual protein translation with TransDecoder v.3.0.1 (http://transdecoder.github.io), using a minimum predicted protein length of 100 amino acids. All the sequences were annotated with the Trinotate pipeline v.3.0 (https://trinotate.github.io) and assigned Gene Ontology categories [31] based on positive BLASTp and BLASTx [28] matches against the UniProtKB/Swiss-Prot database (e-value threshold, 1E^−5^). The annotation of Pfam conserved protein domains [32] was based on the identification by Hmmer v.3.1b2 [33]. In specific cases where no annotation could be assigned based on these criteria, remote structural similarities with templates deposited in the Protein DataBank were inspected with HHpred [34].

### Gene expression analysis

Trimmed reads for each of the five tissues were mapped on the annotated transcriptome with the RNA-seq mapping tool of the CLC Genomics Workbench v.9.5 (Qiagen, Hilden, Germany) using the following parameters: length fraction: 0.75; similarity fraction: 0.98; maximum number of matching contigs: 10; paired-end reads distance was automatically estimated. The number of matched reads were converted into Transcripts Per Million (TPM) expression values [35], a measure which ensures efficient data normalization and comparability both within and between samples. The obtained TPM values, representing the average expression levels of a pool of five biological replicates, were subjected to a statistical analysis of gene expression with a Kal’s Z-test [36]. In detail, the expression profile of a given tissue was compared with the other four to identify differentially expressed genes (DEGs). The thresholds of fold change and False Discovery Rate-corrected *p*-value were set at 2 and 0.05, respectively. All DEGs exceeding these thresholds in all the four comparisons were marked as “tissue-specific”. TPM expression were processed by log_2_ transformation for visualization purpose to generate scatter and volcano plots. A gene expression heat map was created by Euclidean distance-based hierarchical clustering (with average linkage) of a representative set of genes whose expression exceeded 3000 TPM in any of the five tissues taken into account in this study. Heat maps were also similarly generated for genes encoding lectins.

Tissue-specific genes were subjected to a functional evaluation, assessed through hypergeometric tests on Gene Ontology and Pfam annotations [37], implemented in the CLC Genomics Workbench v.9.5. Significantly over-represented annotations were detected based on a *p*-value <0.01 and observed – expected value > = 5.

### Validation by real-time PCR

The expression data obtained by in silico analyses as detailed in the section above was validated by Real-Time PCR on three individual adult mussel, collected and dissected as previously described. Extracted RNAs were used to synthetize cDNA with a qScript™ Flex cDNA Synthesis Kit, QuantaBio (Quanta BioSciences Inc., Gaithersburg, MD) following manufacturer’s instructions. Ten target genes (two for each tissue) were selected for validation among those displaying high tissue specificity in RNA-seq experiments. The primer sequences, designed with Primer3Plus (https://primer3plus.com/) to obtain amplicons of 100–150 nt size, are reported in Table 1. In addition, we selected two stable housekeeping genes, the elongation factor 1 alpha and the ribosomal protein S2 (the former from literature data [38, 39], the latter due to its constant TPM values), to normalize gene expression data among samples.

**Table 1:** Primers used for real-time PCR validation

target gene	forward primer (5′ - > 3′)	reverse primer(5′ - > 3′)
Chitotriosidase	TACTGCGATTGGCCATACAA	TGCCTGTGTAGTTCGAGGTG
Meprin A	GCTTGGGTACATCGACCACT	CTGCTTTGGTTCCAGTGTCA
Paramyosin	CCGAACTCGCAGAAAAGAAC	CGTAGAGCTTCCTCGGTGTC
Myosin, striated muscle	CTCTTGTTGCCCCAGGATTA	CTGGTAGCTCCACCAGCTTC
Acetylcholine receptor	GTCAAAGTCGGCCACTCACT	GTCTGACCGTCGTTGGTTCT
Glycine-rich secreted protein	CACACGGTCTTACTGGAGCA	GTTCGCCTTGTTCACCTTGT
Valine-rich secreted protein	AAAGTGCCATTCGAGACACC	GGGCTGGGAACTCTGAATTT
SCP domain containign protein	TCAAACACTGCGTCAAGACC	GGATCCACGTTTTCTCTTGC
Serine protease inhibitor	AGGCCAACTGCAAAAACG	CCGTCAACTCCACACACG
Tyrosinase-like protein 2	CAGAGCCCTACCTCCAGATG	TGACTGCTCGCTTTGTATGG
EF1alpha	CTCTTCGTCTCCCACTCCAG	ACCAGGGAGAGCTTCAGTCA
Ribosomal protein S2	GCCATTGCCAATACCTATGC	GCCTGGTTGACGAGTATGGT

In detail, PCR reactions were prepared as follows: 5 μL of SsoAdvanced SYBR Green Supermix (Bio-Rad, Hercules, CA), 0.2 μL of the 10 μM forward and reverse primers, 1 μL of 1:20 diluted cDNA and water to reach a final reaction volume of 10 μL. Polymerase chain reaction (PCR) amplifications were carried out on a Real-Time C1000-CFX96 platform (Bio-rad), using the following thermal profile: 95° for 30 s, by 40 cycles at 95° (10 s) and 60° (20 s). The absence of non-specific amplification products was assessed with a melting curve analysis (65° to 95°). Gene expression values for the target genes were calculated using the delta Ct method and normalized on the average expression level of the two housekeeping genes. Results are shown as the average plus standard deviation of three technical replicates.

### Phylogenomic analysis

The phylogenomic analysis was carried out based on a set of 445 single-copy orthologous genes present in the transcriptomes of *M. virgata* and 8 other mytiloid bivalve mollusk species, using the same strategy previously used by Biscotti and colleagues [25]. Namely, the species selected for this purpose were: *Bathymodiolus azoricus*, *Bathymodiolus manusensis*, *Bathymodiolus platifrons*, *Geukensia demissa*, *Lithophaga lithophaga*, *Modiolus kurilensis*, *Modiolus modiolus*, *Modiolus philippinarum*, *Mytilus californianus*, *Mytilus coruscus*, *Mytilus galloprovincialis* (as a representative of the *M. edulis* species complex), *Perna viridis* and *Perumytilus purpuratus*. The full set of the proteins encoded by the recently published genomes of *B. platifrons* and *M. philippinarum* were recovered. For all the other species, publicly available raw sequencing data was downloaded from the NCBI SRA database, imported in the CLC Genomics Workbench 9, trimmed based on quality as described above and assembled with the de novo assembly tool, setting both the *word size* and *bubble size* parameters to “automatic”. Coding sequences (CDSs) were then predicted with TransDecoder v.3.0.1 (http://transdecoder.github.io), as described above for *M. virgata*. Sequence data from the genome of the Pacific oyster *Crassostrea gigas* v.9 [40] were also included to provide a reliable outgroup species for the analysis, based on the recent identification of Ostreoida as a sister group to Mytiloida [41].

Following this procedure, reciprocal BLASTp searches were performed, between the target species, *M. virgata* and *C. gigas* (the outgroup), using an e-value threshold of 1 × 10^−10^; taking into account only the best BLAST hit and discarding all the sequences lacking significant homology in any of the comparisons (either because of an e-value lower than the threshold or because of sequence identity <50%). This finally allowed the identification of a set of conserved orthologous sequences, which were aligned with MUSCLE [42] and further processed with Gblocks v.0.91b to remove highly divergent regions or fragments missing in one or more species due to the incompleteness of transcriptome data [43]. The resulting trimmed alignments were then concatenated and used as an input for a ProtTest v.3.4 analysis [44] in order to identify the best-fitting model of molecular evolution based on the corrected Akaike Information Criterion [45]. The concatenated multiple sequence alignment file, consisting of 247 orthologous proteins unambiguously detected in all the species taken into account, was subjected to Bayesian phylogenetic inference analysis with MrBayes v.3.2 [46], based on the LG model of molecular evolution, with a Gamma-shaped distribution of rates across sites, a proportion of invariable sites and fixed (empirical) priors on state frequencies (LG + G + I + F), identified by ProtTest as the best-fitting model. Phylogenetic inference was carried out with two independent analyses run in parallel. The convergence of the parameters generated by the two MCMC analyses was evaluated with Tracer v.1.6 (http://beast.bio.ed.ac.uk/Tracer). The analysis was stopped when the Effective Sample Size of each estimated parameter reached a value higher than 200, without considering the initial 25% of the generated trees (removed due to the burnin process). Sampled trees were used to calculate a consensus phylogenetic tree, where poorly supported nodes (those displaying posterior probability <50%) were collapsed.

## Results and discussion

### The high quality transcriptome of *Mytilisepta virgata*

Following the application of quality filters, the de novo transcriptome assembly of *M. virgata* comprised 49,501 contigs with an average length of 679 nucleotides (Table 2). This number, apparently rather high if compared to the number of annotated genes in bivalve genomes (e.g. ~26,000 in *C. gigas* and ~33,000 in *P. fucata*) [40, 47], is possibly justified by at least three factors: (i) the poor knowledge of bivalve non-coding RNAs, that are abundant in mussels [14], including in *M. virgata*, where they account for 71% of the assembled contigs; (ii) the partial fragmentation of mRNAs in smaller contigs; (iii) the high genomic complexity and heterozygosity of the genome of mytiloids, previously pointed out in *Mytilus* spp. [16].

**Table 2:** Sequencing, de novo assembly and annotation statistics

Digestive gland trimmed reads	48,106,544
Posterior adductor muscle trimmed reads	46,456,019
Gills trimmed reads	49,760,348
Mantle rim trimmed reads	53,492,730
Foot trimmed reads	86,035,069
Number of contigs	49,501
Longest contigs	9778 nt
Average contig length	679 nt
N50	1046
Contigs longer than 5Kb	68
Complete BUSCOs	66%
Fragmented BUSCOs	10%
Absent BUSCOs	24%
Predicted protein-coding contigs (>100 aa)	29%
GO cellular component annotation rate	19%
GO molecular function annotation rate	17%
GO biological process annotation rate	17%
PFAM annotation rate	23%
Mytiloid innovations (protein coding)	2%

**Fig. 2:**
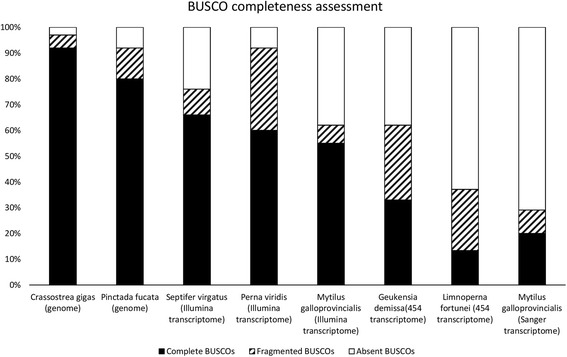
Comparison of transcriptome completeness, estimated with Busco v.2, among publicly available de novo assembled transcriptomes from Mytiloida and gene model predictions from the fully sequenced genomes of *Crassostrea gigas* and *Pinctada fucata*

N50, the metric most commonly used to assess the quality of a de novo assembly [48] was 1046, in line with those obtained in the de novo assembly of other species of the order Mytiloida using Illumina sequencing technologies [14, 15]. While mean contig length (679 nt) and N50 metrics can only provide a measure of the effectiveness of the de novo assembly pipeline, the completeness of a transcriptome can be only theoretically estimated through the comparison with its reference genome. However, a similar estimate can be performed also when a reference genome is not available, using a set of highly conserved single-copy orthologous sequences. These genes, expected to be found across all the genomes within a target taxa, can be defined as “Benchmarking Universal Single-Copy Orthologs” (BUSCOs) [29]. This analysis revealed that ~66% of the conserved metazoan orthologous genes was present and complete in the assembled transcriptome of *M. virgata*, whereas only ~10% were present but fragmented, and ~24% were absent (Fig. 2). This result highlights a slightly higher level of completeness of the *M. virgata* transcriptome compared to other mussel transcriptomes obtained with Illumina sequencing technologies from single tissues [14, 49], much higher than those obtained with more error-prone or lower throughput technologies (454 Life Sciences and Sanger sequencing) [18, 50, 51] and just slightly lower than bivalve genome-scale protein prediction in oysters [40, 47]. This supports our choice of combining RNA-seq from multiple tissues and different specimens to obtain a representative nearly-complete transcriptome. The residual incompleteness of the de novo assembled transcriptome, evidenced by the missing BUSCOs, can be explained by the fact that some genes were not expressed at all in the five tissues taken into account. Specifically, as no transcriptome from larval stages was sequenced in this study, genes related to embryonic development might have been entirely missing. At the same time, since inner mantle (invaded by gonads during the spawning season) and hemocytes could not be analyzed due to insufficient RNA quality, a number of genes displaying high specificity of expression in these two highly specialized tissues are likely to be absent in the assembled transcriptome. Finally, tightly regulated genes whose expression is turned on in response to particular stimuli are also expected to be absent due to naïve condition of the mussel specimens collected.

Overall, poor expression can also lead to contig fragmentation, together with other factors such as transcriptome complexity, heterozygosity and inter-individual variability. Taking into account that mussels pertaining to the genus *Mytilus* display an astounding level of heterozygosity and are subject to significant genetic introgression [52], the relatively low rate of fragmented/complete BUSCOs (0.15) was somewhat unexpected. Since no information is currently available concerning the genetic diversity of *M. virgata* populations, these results possibly suggest that the mean level of heterozygosity in this species is significantly lower than that found in *Mytilus* spp., which would be consistent with a smaller effective population size.

The annotation rate by Trinotate was quite low, as only about 25% of the assembled contigs could be associated to a Gene Ontology term or to a Pfam domain. This observation, which is in line with previous results obtained from transcriptome studies in other mytiloids [14], is linked to at least three different factors: (i) partial fragmentation of contigs, as evidenced by BUSCO; (ii) high prevalence of taxonomically restricted genes; (iii) high prevalence of non-coding transcripts.

In detail, the presence of gene families restricted to Mytiloida has been previously described [53, 54] and, while this topic should be better investigated once the first complete mussel genome will become available, a brief analysis revealed that ~2% (1078) of the assembled sequences pertained to gene families exclusively found in mytiloids (as evidenced by the lack of BLAST homology with non-mytiloid bivalve genomes and transcriptomes, as opposed to significant homology against the publicly available genomes and transcriptomes of other mytiloids).

At the same time, the universe of non-coding RNAs in invertebrates remains to be fully investigated. While genome annotation pipelines, in most cases, currently disregard non-coding genes in newly assembled invertebrate genomes due to lack of homology and experimental evidence, the number of non-coding genes annotated in the genome of the model organism *Caenorhabditis elegans* has recently surpassed that of coding genes, starting to unveil the important role these RNAs cover in the biology of protostomes. Consistently with this observation, only 29% of the *M. virgata* contigs were found to contain an ORF longer than 100 codons, suggesting that a high fraction of non-coding sequences was indeed the primary reason of the low annotation rate.

Complete annotation data and gene expression levels are provided in Additional file 1.

### Phylogenetic relationship with other mytiloids

The phylogenetic position of *M. virgata* and the classification of this species within the *Septifer* (Récluz, 1848) or the *Mytilisepta* (Habe, 1951) genus have been a matter of debate. Currently, although *Septifer virgatus* and *Mytilisepta virgata* are considered as synonyms by the World Register of Marine Species and by MolluscaBase [55], only the latter is marked as an accepted species name. Based on the same taxonomy reference databases, the genus *Mytilisepta* currently includes two other species, *M. bifurcata* (Conrad, 1837) and *M. keenae* (Nomura, 1936). On the other hand, nine different species are currently recognized as pertaining to the *Septifer* genus by this taxonomical authority. However, the *Mytilisepta* genus is currently not accepted by the NCBI taxonomy authority and *M. virgata* is therefore still listed as *S. virgatus* and further placed within Mytilinae subfamily.

The traditional classification of both *Mytilisepta* and *Septifer* species as members of the same genus is strictly based on morphological features, namely the presence of a septum which is involved in the attachment of the adductor muscle to the shell. However, recent molecular phylogenetic studies based on the analysis of 18S/28S nucleotide sequence have strongly suggested that the presence of this structure in the two mussel genera is the result of convergent evolution. Indeed, *M. virgata* and *S. bilocularis* pertain to highly divergent clades, with the former being related to mussel species pertaining to the Brachidontinae subfamily [9].

Taking advantage from the large amount of sequence data generated in the present study and transcriptomic dataset publicly available for other mytiloid species, we inferred the phylogenetic placement of *M. virgata* within the order Mytiloida by the use of Bayesian methods. Our phylogenomics approach, which took into account a set of 247 single-copy orthologous genes detected in all the studied species, is expected to provide a highly reliable estimate of the evolutionary relationship of *M. virgata* with other mussel species, as recently demonstrated by a series of phylogenomics studies which have helped to discern the phylogenetic relationship of Mollusca and Bivalvia [41, 56–58].

Before proceeding to the discussion of the placement of *M. virgata*, it is worth to briefly remind that the classical classification of mytiloids, currently used by NCBI Taxonomy, comprises seven subfamilies: (i) Bathymodiolinae, giant deep see mussels associated with hydrothermal vents; (ii) Brachidontinae (Nordsieck, 1969), small-sized “scorched mussels” adapted to life in the mid-intertidal zone; (iii) Crenellinae (Gray 1840), small-size byssal nets-creating benthic species commonly found in sandy and muddy seabeds; (iv) Lithophaginae (H. Adams & A. Adams, 1857), rock- or coral-boring mussels with a cylindrical, elongated shape; (v) Modiolinae (G. Termier & H. Termier, 1950), presenting a rounded outline and subterminal umbones, which usually live buried in the soft sediments of the subtidal zone; (vi) Mytilinae (Rafinesque, 1815), usually presenting a triangular shape and terminal umbones and commonly creating dense mussel beds in the intertidal zone of rocky shores; (vii) Septiferinae, previously including also *M. virgata* and other morphologically similar mussel species with am arcuate and convex anterior margin of the shell.

**Fig. 3:**
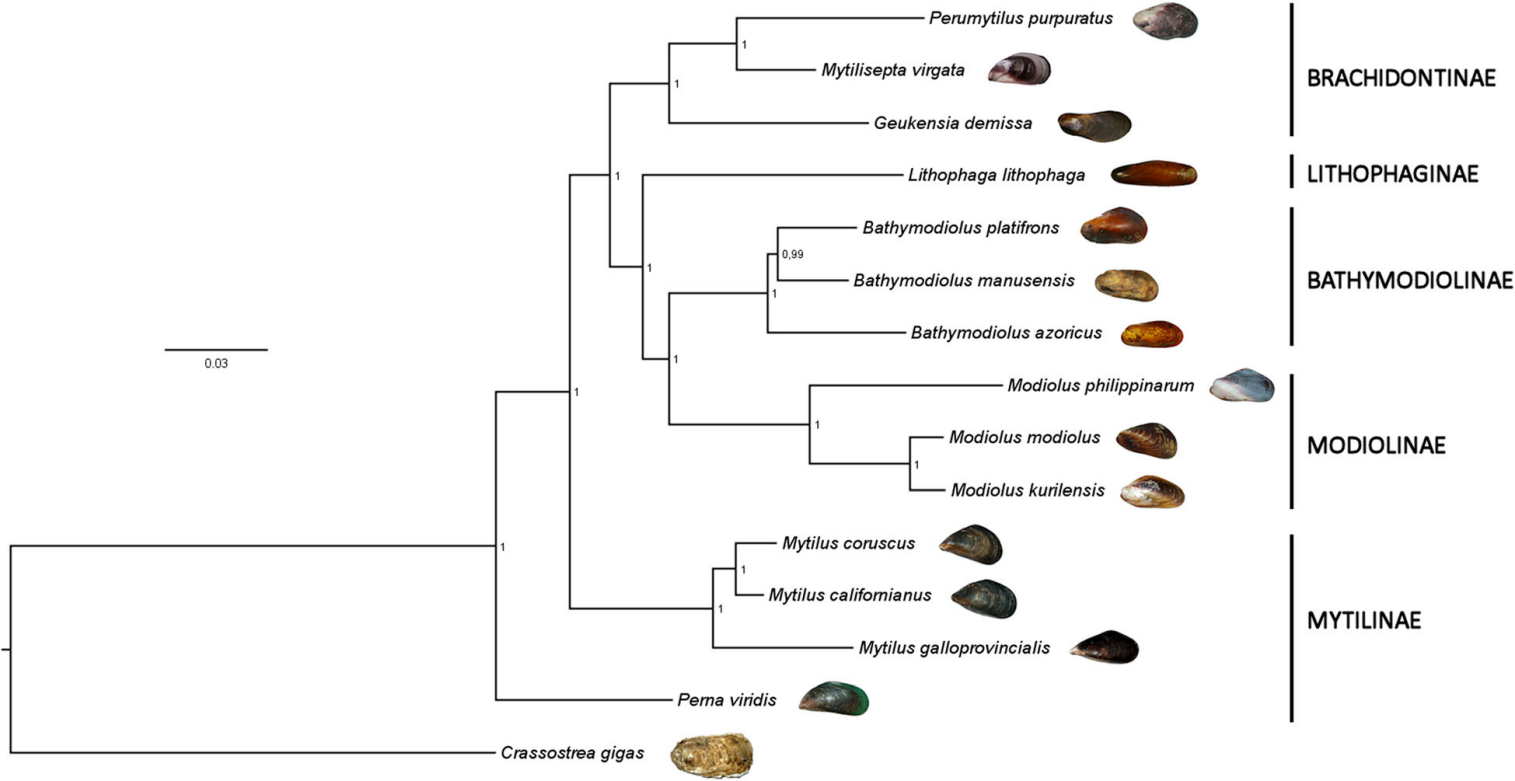
Bayesian phylogeny of Mytiloida. The classical taxonomical classification of mytiloids, according to NCBI Taxonomy, is shown on the right side of the figure. No species pertaining to the subfamilies Crenellinae and Septiferinae could be included due to the absence of available sequence data. Note the placement of *M. virgata* within the Brachidontinae subfamily. The number indicated on the nodes of the tree indicate posterior probability support values

The phylogenetic tree obtained was highly supported in all its nodes by posterior probability values higher than 99%, indicating the nearly certain and unambiguous inference of the most likely evolutionary scenario which led to the radiation of mussels (Fig. 3). Remarkably, *M. virgata* was found to be closely related to *P. purpuratus*, supporting the results previously published by Trovant and colleagues based on ribosomal RNA sequence data [9], and clearly placed within the Brachidontinae (Nordsieck, 1969) subfamily, together with the ribbed horsemussel *G. demissa*. While no –omic scale sequence data is available for other species currently classified in the *Septifer* genus, molecular phylogeny produced by other authors suggest that *S. excisus*, *S. bilocularis* and other congeneric species are distantly related to *M. virgata* and do not belong to Brachidontinae, being more closely related to *Mytilus* (Linnaeus, 1758) and other species of the subfamily Mytilinae.

Based on these results, the current official naming of *M. virgata* as *S. virgatus* at the NCBI taxonomy database does not appear to be appropriate, and it should be updated following the official nomenclature already adopted by WoRMS and MolluscaBase. Moreover, *Mytilisepta* appears to be clearly pertaining to the Brachidontinae subfamily, which at the present time only lists the *Brachidontes* (Swainson, 1840), *Geukensia* (Van der Poel, 1956), *Ischadium* (jules-Brown, 1905) and *Perumytilus* (Olsson, 1961) genera in the NCBI taxonomy database.

### Overview of gene expression profiles

**Fig. 4:**
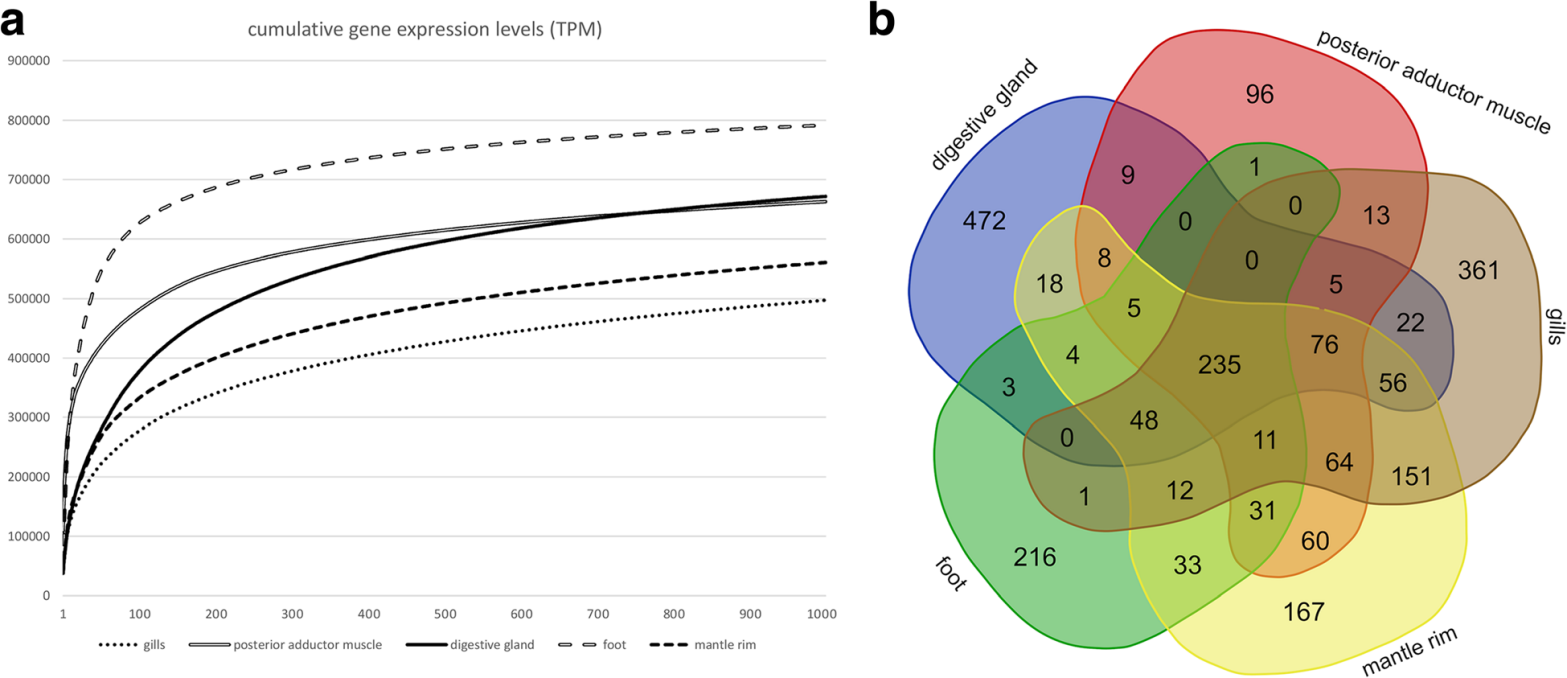
Panel (**a**) cumulative gene expression of the 1000 most expressed genes per tissue. The gene expression level on the Y axis is measured as TPM. Panel (**b**) Venn diagram displaying the overlap between highly expressed transcripts (TPM > 100) among the five tissues subjected to RNA-seq

As an overall trend, the five analyzed tissues displayed widely diverse gene expression profiles, characterized by a broad prevalence of tissue-specific genes (Fig. 4). Cumulative expression plots revealed that one of the major differences among tissues can be attributed to the high energetic investment in the synthesis of a very few mRNA species by some tissues (e.g. foot) compared to a more even distribution of the transcriptional efforts by other tissues (e.g. gills). This factor can be efficiently estimated by the relative contribution to global transcriptional activity of the 100 most highly expressed genes in any given sample. We define the reciprocal of this value as *Transcriptomic Diversity Index* (TDI). Therefore, a high TDI indicates the expression of a broad range of transcripts, whereas a low TDI points out a low diversity in the mRNA population. The TDI values calculated for the *Mytilisepta* tissues were, from the highest to the lowest: 3.57 (gills), 3.03 (mantle rim), 2.63 (digestive gland), 2.08 (posterior adductor muscle) and 1.59 (foot) (Fig. 4, panel a). As a further confirmation of the remarkable functional specialization of bivalve tissues, just a relatively low number of genes (235), mostly encoding fundamental housekeeping genes, were found to be expressed at relatively high levels (TPM > 100) in all the five tissues analyzed (Fig. 4, panel b).

**Fig. 5:**
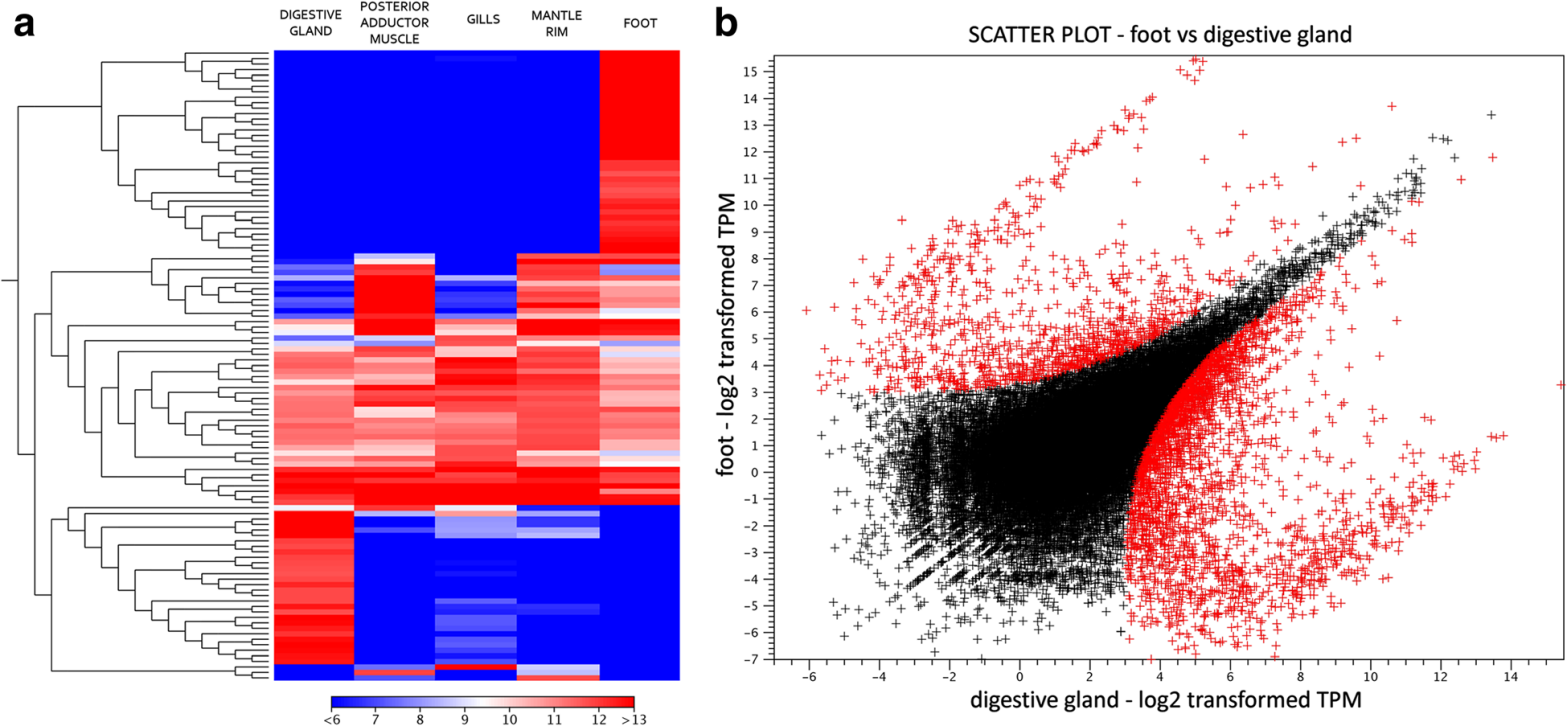
Panel (**a**) Euclidean distance-based hierarchical clustering, calculated based on average linkage, of the *Mytilisepta virgata* transcripts reaching an expression level higher than 3000 TPM in at least on out of the five tissues analyzed. Panel (**b**) Gene expression scatter plot comparing the expression profiles of digestive and foot in *Mytilisepta virgata.* The log_2_ normalized TPM gene expression levels are reported on the X and Y axes. Differentially expressed genes are marked in *red*

The functional differentiation of the five mussel tissues appears to be evident from the hierarchical clustering of genes based on their pattern of expression (Fig. 5, panel a). The digestive gland and the foot, in particular, are characterized by two clusters of genes with high tissue specificity, nearly undetectable outside the main site of production. At a first sight, the hierarchical clustering analysis does not permit to clearly identify equally important differences among mantle rim, gills and posterior adductor muscle. However, while these differences are not as evident as those observed for foot and digestive gland, they might influence in a highly significant manner the function of a tissue (see the following sections).

### Digestive gland transcriptional profile

The digestive gland is the main tissue involved in the digestion of food particles, in the conjugation of nutrients to carriers for their transportation through circulation, in the metabolism of xenobiotics and in the excretion process. In addition, it also covers an important role in the long-term storage of nutrient reserves to be used during gametogenesis or long-lasting stress periods. Two main cell types are responsible for the fundamental functions of this organ, i.e. digestive cells and basophil secretory cells. These cells are located in the blind end of digestive tubules branching from the main digestive ducts. Here, highly abundant columnar digestive cells adsorb food particles by pinocytosis and proceed to their digestion upon the fusion of endocytotic phagosomes with lysosomes. On the other hand, pyramid-shaped secretory cells are thought to be mainly involved in the release of enzymes involved in the extracellular breakdown of major food particles [59].

Mussels feed on a wide variety of food particles found in the water column, including phytoplankton, micro-zooplankton, bacteria and detritus, whose abundance largely varies both seasonally and geographically. For this reason, mussels display limited food preference, mostly based on size selection [60], and they actively produce of a broad range of digestive enzymes required for the assimilation of diverse nutrients, i.e. fat, carbohydrates and proteins found in different food sources.

Coherently with these observations, the expression profile of the *M. virgata* digestive gland was typical of a highly specialized tissue, displaying tissue-specificity for 2043 genes, as evidenced by the expression scatter plots (Fig. 5, panel b) and a low TDI (2.63). The transcriptional profile was dominated, as expected, by digestive enzymes. The gene set enrichment analysis indeed clearly highlighted that a major energetic effort is spent in the synthesis of enzymes involved in the processing of the two most abundant biomasses in nature, cellulose and chitin [61], i.e. cellulases (identified as members of the glycosyl hydrolase families 9 and 10) and chitinases (pertaining to the glycosyl hydrolases family 16) (Table 3). The overwhelming presence of these classes of enzymes among the most highly expressed genes is probably ascribable to the wide availability of micro-zooplankton and microalgae as potential food sources in the oceans.

**Table 3:** The 25 most highly expressed genes in *Mytilisepta virgata* digestive gland and representative significantly over-represented annotations by gene set enrichment analysis

Most expressed genes		Over-represented annotations		
Annotation	TPM	Term	Class	*p*-value
Vdg3	43,813.51	Extracellular space	CC	0.00
Probable apolipoprotein	14,067.11	ShK domain-like	PFAM	0.00
Probable apolipoprotein	12,230.17	Ependymin	PFAM	0.00
Alpha tubulin	11,375.80	Lectin C-type domain	PFAM	1.11E-16
Elongation factor 1 alpha	11,074.06	C1q domain	PFAM	2.22E-16
Ependymin-related protein	11,054.83	von Willebrand factor type C domain	PFAM	1.07E-14
Ependymin-related protein	8275.80	Cellulose catabolic process	BP	3.83E-12
Vdg3	8084.66	Lysosome	CC	7.27E-12
Chitotriosidase	7926.27	Cellulase activity	MF	4.21E-11
Unknown	6439.28	Glycosyl hydrolase family 9	PFAM	8.40E-11
Probable apolipoprotein	6230.41	alpha/beta hydrolase fold	PFAM	2.00E-10
Cytoplasmic actin	6103.04	Carboxylesterase family	PFAM	3.77E-10
Calcium binding protein	5887.19	Trypsin	PFAM	6.07E-10
Meprin A	5695.11	Glycosyl hydrolases family 16	PFAM	8.76E-9
60S ribosomal protein P0	5364.73	Serine-type endopeptidase activity	MF	1.34E-8
Ependymin-related protein	5342.49	MAM domain, meprin/A5/mu	PFAM	2.76E-8
Urokinase-like protein	5283.92	Carbohydrate metabolic process	BP	5.91E-8
Ganglioside GM2 activator	5163.45	Lipid transporter activity	MF	8.20E-8
Putative metalloprotease	4892.49	Antistasin family	PFAM	1.36E-7
Alpha tubulin	4698.32	Sulfatase	PFAM	1.17E-6
Spondin-like protein	4503.80	Glycosyl hydrolase family 10	PFAM	5.83E-6
40S ribosomal protein SA	4288.17	Peptide metabolic process	BP	4.26E-6
C1q domain-containing protein	4279.26	Lipase	PFAM	8.19E-6
Ependymin-related protein	3725.69	Prolyl oligopeptidase family	PFAM	8.39E-5
Calcium binding protein	3528.24	Xenobiotic metabolic process	BP	4.56E-4

See the complete list in Additional file 4

*TPM* Transcript Per Million, *PFAM* Pfam conserved domains, *BP* Gene Ontology Biological Process, *MF* Gene Ontology Molecular Function, *CC* Gene Ontology Cellular Component

At the same time, fat-digesting enzymes (lipases, in particular) and proteases were also found to be expressed at highly significant levels. The latter include trypsin-, meprin- and papain-like proteinases, cathepsins, several serine-type endopeptidases and a large family of putative metalloproteases characterized by the ShK domain [62]. The high expression of protease inhibitors, such as antistasin, targeting trypsin [63], and Kazal-type serine-protease inhibitors [64], is also in line with the strong production of the aforementioned proteolytic enzymes, whose functional dysregulation could lead to serious cellular and tissue damage. Altogether, while some of these annotations are likely linked to extracellular digestive processes, others clearly point towards lysosomes, which are found in high numbers in vacuolated digestive cells. Among these, sulfatases and of ganglioside GM2 activator, highly expressed in the digestive gland, are probably the most relevant. The former is involved in the lysosomal cleavage of sulfated carbohydrates; the latter is a cofactor for the lysosomal digestion of gangliosides.

As a result of its pronounced endocytotic activity, the digestive gland is also a main site of bioaccumulation for pollutants, heavy metals and biotoxins [65, 66], which are processed by detoxifying enzymes. The specialization of the digestive gland in this activity is indeed evidenced by the overrepresentation of oxidoreductases and carboxylesterases, phase I detoxifying enzymes which introduce polar groups in xenobiotics to increase their solubility and promote their excretion.

Furthermore, consistently with the function homologous to that covered by liver in vertebrates, a few highly expressed genes in the digestive gland of *Mytilisepta* encode apolipoproteins, devoted to the binding and transportation of lipids in the circulatory system. In particular, the second and the third most highly expressed genes (Table 3), despite lacking detectable primary sequence similarity, display a remarkable predicted structural similarity with apolipoproteins, identified by HHpred as detailed in the materials and methods section.

Similarly to what has been previously reported in *M. galloprovincialis* [14, 67], the most highly expressed gene in the digestive gland of Japanese mussels was vdg3, a developmental marker of the maturation of this organ in juveniles [68], whose precise biological function still remains to be fully elucidated. Vdg3, which is probably encoded by a few highly similar paralogous genes (like in the Mediterranean mussel [69]), accounts for more than 4% of the total transcriptional activity of the *M. virgata* digestive gland.

In addition, it is worth of note that the digestive gland, besides being involved in the secretion of enzymes for extracellular digestion, also releases in the extracellular environment a large number of proteins with potential carbohydrate binding properties, most notably C-type lectins and C1q domain-containing (C1qDC) proteins (Table 3; see the following sections for a detailed overview on these gene families).

### Foot transcriptional profile

The foot is an important tissue both in the larval and in the adult phases of mussel life. Although the foot has progressively lost its ancestral function as the main locomotory organ along bivalve evolution, it still retains a limited role in the movement of pediveliger larvae and juvenile mussels. In adult specimens, the foot is the main responsible of the attachment to suitable substrates through the secretion of byssal threads, robust and elastic fibers produced by a specialized secretory glands, which have attracted a considerable interest due to their potential biotechnological applications [70].

The gene expression profile (TDI = 1.59, see Fig. 4 panel a) pointed out that the foot is by far the most specialized tissue among those subjected to RNA-seq and the one producing the lowest variety of transcripts. As evidenced by gene set enrichment analyses, most of the 769 foot-specific transcripts identified in *M. virgata* were indeed clearly associated with the production of byssus. In particular, ~15% of the total transcriptional activity was used for the production of collagens and ~13.5% for the production of byssal cuticle proteins (Table 4). Precollagens are the major constituents of the fibrous core of byssal threads, contribute to their self-assembly and account for more than 70% of their mass [71, 72]. On the other hand, the astounding resistance of these threads is provided by a stiff coating by proteins rich in modified amino acids, such as trans-4-hydroxyproline, trans-2,3-cis-3,4-dihydroxyproline, and L-3,4-dihydroxyphenylalanine (L-DOPA), which form a 2–5 μm thick highly resistant cuticle [73]. We found a significant sequence similarity between the byssal cuticle proteins of *M. virgata*, those of the deep sea vent mussel *Bathymodiolus thermophilus*, and an adhesive polyphenolic foot protein of *Septifer bifurcatus*. These low-complexity proteins in fact consist of a high number of consecutive repeats of conserved 17-mers, which present, in all the three species, a well detectable Y-X-X-X-Y-K motif required for adhesion [74] (Additional file 2). Besides these two classes of sequences, the gene expression profile of the foot was dominated by other low-complexity proteins, including YGH-rich proteins similar to those previously identified in *Mytilus coruscus* [75], and proteins containing EGF-like domains, which characterize cross-linking mussel adhesive plaque proteins [76].

**Table 4:** The 25 most highly expressed genes in *Mytilisepta virgata* foot and representative significantly over-represented annotations by gene set enrichment analysis

Most expressed genes		Over-represented annotations		
Annotation	TPM	Term	Class	*p*-value
Byssal cuticle protein	45,262.97	Collagen triple helix repeat (20 copies)	PFAM	0.00
Byssal cuticle protein	43,379.14	Serine-type endopeptidase inhibitor activity	MF	0.00
Collagen-like protein	42,699.96	Negative regulation of coagulation	BP	1.11E-16
Byssal cuticle protein	34,293.81	Common central domain of tyrosinase	PFAM	3.33E-16
Collagen-like protein	33,886.43	Extracellular region	CC	3.33E-16
Serine protease inhibitor	30,056.14	Negative regulation of endopeptidase activity	BP	6.77E-15
Low complexity protein	26,250.15	Animal haem peroxidase	PFAM	6.77E-14
Collagen-like protein	17,017.56	Kazal-type serine protease inhibitor domain	PFAM	4.35E-12
YGH-rich protein-2	16,065.78	Peroxidase activity	MF	5.69E-12
Collagen-like protein	15,351.63	Proteinaceous extracellular matrix	CC	6.47E-12
Collagen-like protein	13,389.57	Tissue inhibitor of metalloproteinase	PFAM	6.83E-10
Byssal cuticle protein	12,178.71	Collagen trimer	CC	2.14E-8
Collagen-like protein	10,965.75	Response to oxidative stress	BP	5.45E-8
Elongation factor 1 alpha	10,722.32	Regulation of keratinocyte differentiation	BP	8.93E-8
Collagen-like protein	10,461.88	Hydrogen peroxide catabolic process	BP	1.05E-7
YGH-rich protein-1	10,019.89	Transforming growth factor beta binding	MF	1.63E-7
Precollagen NG	9810.75	Metal ion binding	MF	8.77E-7
YGH-rich protein-1	8119.26	Extracellular space	CC	9.20E-7
Serine protease inhibitor	7406.30	Heme binding	MF	2.55E-6
Serine protease inhibitor	7362.32	Oxidoreductase activity	MF	6.45E-6
Collagen-like protein	7349.68	WAP-type ‘four-disulfide core’	PFAM	1.01E-5
Collagen-like protein	7307.02	Quinone binding	MF	1.26E-5
Unknown	7104.95	Human growth factor-like EGF	PFAM	1.78E-5
Kazal-type serine proteinase inhibitor	7095.67	EGF-like domain	PFAM	6.84E-5
Low complexity protein	6445.09	Kunitz/Bovine pancreatic trypsin inhibitor domain	PFAM	7.62E-5

See the complete list in Additional file 4

*TPM* Transcript Per Million, *PFAM* Pfam conserved domains, *BP* Gene Ontology Biological Process, *MF* Gene Ontology Molecular Function, *CC* Gene Ontology Cellular Component

Interestingly, tyrosinases were the most important class of enzymes expressed in this tissue: 13 out of the 18 tyrosinase sequences found in the purplish mussel transcriptome were indeed specifically expressed in the foot. This data is in agreement with the importance of this enzymatic activity in the conversion of tyrosine residues to L-DOPA and, consequently, to dopaquinone in Tyr-rich byssal cuticle proteins [77, 78]. At the same time, the high observed activity of antioxidant enzymes (peroxidases in particular) is explained by the need of maintaining a redox balance to allow the modification of L-DOPA-rich adhesion proteins [79, 80].

Consistently with previous reports, many proteinase inhibitors were selectively expressed at high levels in the foot. Among these, the strong expression of serine-type endopeptidases and alpha-2 macroglobulins is certainly worth of a note. Moreover, the foot-specificity of six WAP-like proteins suggest that they may act as suppressors of elastase-type serine proteases, preventing laminin degradation [81]. These enzyme inhibitors may altogether constitute an efficient protecting system that prevents the degradation of byssal threads by extracellular proteases [75].

### Gills transcriptional profile

In all non-protobranch bivalves, gills are large organs whose structure follows the curvature of the shell, dividing the mantle cavity between inhalant and exhalant chambers and providing a large surface area exposed to the incoming water flow. Their lamellar structure, strengthened by collagen, makes them well suited for both gas exchange and filter-feeding. Gills are highly vascularized by vessels which bring deoxygenated hemolymph to the filaments, where the gas exchange takes place, and subsequently recirculate oxygenated fluid to the whole body through an efferent vein. The feeding function on the other hand is carried out by specialized cilia that cover the entire branchial surface and convey food particles carried by inflowing water towards the labial palps. Overall, gills are very complex organs that combine functionally and structurally diverse cell types, including neuronal cells that depart from a visceral ganglion.

Dissimilarly from other highly specialized tissues (i.e. foot and digestive gland), gills do not show the expression of a well-evident group of highly tissue-specific genes in the scatter plots (Additional file 3). As mentioned above, this is possibly linked to the diversity of cell types present in this complex organ, resulting in a partial overlap with the transcriptomes of the other four tissues analyzed. As a matter of fact, gills represent the richest out of the five tissues in terms of transcriptional diversity (TDI = 3.57) (Fig. 4, panel a).

In any case, the number of genes whose expression was statistically significantly higher in this tissue compared to the others was rather high (2877). These genes were, for the most part, linked to the movement of motile cilia, the main players in the filter-feeding process, present at very high densities on the whole surface of the gills. In particular the expression of axonemal dyneins, that drive ciliary movement by regulating the relative sliding of microtubule doublets [82], was very prominent, as well as that of tektins, fundamental for ciliar assembly and functionality [83] (Table 5). As the function of dyneins require ATP, mitochondria-eating proteins, significantly overexpressed, may be used to improve mitochondrial function, enhancing energy production [84]. The strong production of several genes encoding neuronal acetylcholine receptor subunits [85] was also likely related to the coordinated movement of cilia. In this context, acetylcholine could lead to a strong enhancement of ciliary beat frequency and Shisa-like proteins, also over-represented, may act as regulators of its trafficking [86].

**Table 5:** The 25 most highly expressed genes in *Mytilisepta virgata* gills and representative significantly over-represented annotations by gene set enrichment analysis

Most expressed genes		Over-represented annotations		
Annotation	TPM	Term	Class	*p*-value
Alpha tubulin	55,938.25	Dynein heavy chain and region D6 of dynein motor	PFAM	1.43E-13
40S ribosomal protein SA	16,641.12	Microtubule	CC	0.00
Elongation factor 1 alpha	11,612.37	Microtubule motor activity	MF	6.20E-14
Alpha tubulin	8853.84	Microtubule-based movement	BP	2.52E-10
Beta tubulin	8710.17	Motile cilium	CC	3.06E-10
Actin	8589.14	Axoneme assembly	BP	1.57E-9
Alpha tubulin	8158.42	Cilium movement	BP	2.22E-9
Low complexity glycine-rich protein	7335.45	Acetylcholine-activated cation-selective channel activity	MF	5.94E-8
Beta tubulin	5932.28	C1q domain	PFAM	3.23E-7
Actin	5614.26	ATP-binding dynein motor region D5	PFAM	1.32E-6
Beta tubulin	5144.94	Tektin family	PFAM	2.15E-6
Beta tubulin	5004.75	Axonemal dynein complex	CC	2.63E-6
Actin	4296.64	Neurotransmitter-gated ion-channel ligand binding domain	PFAM	6.18E-6
60s ribosomal protein P0	4296.41	Dynein complex	CC	1.06E-5
non coding RNA	4137.29	ATPase activity	MF	1.56E-5
Ferritin	3614.02	Shisa/Wnt and FGF inhibitory regulator	PFAM	3.71E-5
Ribosomial Protein S10	3367.63	Microtubule-binding stalk of dynein motor	PFAM	4.65E-5
Cyclophilin B	3364.42	Collagen trimer	CC	5.40E-5
Transcription factor ATF4	3351.91	Immunoglobulin C1-set domain	PFAM	7.45E-5
Beta tubulin	3296.13	Fibrinogen beta and gamma chains, C-terminal globular domain	PFAM	1.44E-4
non coding RNA	3201.67	LITAF-like zinc ribbon domain	PFAM	1.60E-4
Cytochrome c oxidase polypeptide	3059.53	TIR domain	PFAM	2.28E-4
Outer dense fiber protein 3	3038.49	Mitochondria-eating protein	PFAM	2.42E-4
Ribosomal Protein L15	2921.58	Neurotransmitter-gated ion-channel transmembrane region	PFAM	6.71E-4
Ribosomal Protein S4	2769.43	Cadherin domain	PFAM	7.75E-4

See the complete list in Additional file 4

*TPM* Transcript Per Million, *PFAM* Pfam conserved domains, *BP* Gene Ontology Biological Process, *MF* Gene Ontology Molecular Function, *CC* Gene Ontology Cellular Component

The high expression of a number of genes encoding extracellular matrix components is certainly connected with the lamellar organization of branchial filaments, which facilitates gaseous exchanges for the respiratory function. Collagens, cadherins and the over-represented “secreted acidic and rich in cysteine Ca binding region proteins”, corresponding to osteonectin-like glycoproteins, appear to be the most important players in the establishment of these complex morphological structures. No specific domain or annotation related to oxygen binding could be identified as unequivocally linked to gills, probably due to the fact that this function is carried out by circulating cells which are also found in other tissues.

Immunity markers certainly represent one of the most noticeable characterizing gene expression signatures of gills. This may be correlated with the large surface of contact with the external environment and, consequently, with potentially pathogenic microorganisms. Both the gene families of C1qDC and FREPs (discussed in detail in the following sections), lectin-like proteins potentially involved in Pathogen Associated Molecular Patterns (PAMPs) recognition [87, 88], were over-represented in the gills transcriptome of the Japanese purplish mussel. At the same time, some TIR-domain containing proteins were also preferentially expressed in the gills. The TIR domain is found in a number of proteins linked to the detection of foreign ligands and to the ransduction of immune signals inside the cell. In detail, the expression of the cytosolic gene products MyD88, STING and ecTIR-DC families 6, 11 and 13 was particularly elevated in this tissue [89]. Moreover, a number of LITAF-like transcription factors, which may positively regulate the production of pro-inflammatory cytokines [90], were also selectively expressed in the gills.

While these signatures could be, at least in part, linked to the presence of hemocytes carried by hemolymph vessels, these data suggest that the gills of *M. virgata* might play an important role as a first line of defense towards pathogens, specifically by releasing soluble factors in the mucus that covers most of the branchial surface.

### Mantle rim transcriptional profile

In mussels, the mantle is a two-lobed tissue that covers the entire inner face of the shells and hosts gonadal development. During the non-reproductive season however, the mantle becomes a very thin and nearly-transparent tissue, whose main functions are the storage of nutrients and the deposition of the shell. The region close to the edges, external to the pallial line of attachment to the shell, is known as mantle rim; this modified part of the mantle tissue is darkly pigmented and possesses tentacles with sensorial function which can be extruded from the shell when the valves are open. Furthermore, the mantle rim is rich in muscle fibers and nerves that permit the retraction of tentacles upon chemical, physical or light stimuli. The portion of tissue sampled in the present experiment corresponds to the large inhalant syphon that regulates the flux of water to the mantle cavity. In mussels, this modified region of mantle is less specialized than other bivalves, as it does not form an exhalant tube like in burrowing bivalve species.

The gene expression profile of the mantle rim only displayed little specialization, as evidenced by the expression scatter plots (Additional file 3). It was indeed characterized by a remarkable overlap with those of the other tissues and a relatively low number of genes (694) were specifically expressed in this tissue and just a very few out of these were expressed at very high levels (Table 6). Possibly owing to the combination of multiple cell types with widely diverse functions, the mantle rim produced a very broad range of transcripts and was only second to the gills for transcriptional diversity (TDI = 3.03, Fig. 4, panel a).

**Table 6:** The 25 most highly expressed genes in *Mytilisepta virgata* mantle rim and representative significantly over-represented annotations by gene set enrichment analysis

Most expressed genes		Over-represented annotations		
Annotation	TPM	Term	Class	*p*-value
Actin	40,034.02	Proteinaceous extracellular matrix	CC	1.84E-7
Elongation factor 1 alpha	17,159.08	Extracellular region	CC	1.87E-7
alpha tubulin	16,259.80	Chitin binding Peritrophin-A domain	PFAM	4.58E-7
60s ribosomal protein L23	15,108.07	Matrixin	PFAM	1.14E-6
Actin	15,102.97	Calcium ion binding	MF	2.65E-6
40S ribosomal protein SA	11,849.66	WAP-type (Whey Acidic Protein) ‘four-disulfide core’	PFAM	3.65E-6
Actin	11,356.99	Extracellular matrix structural constituent	MF	3.66E-6
Alpha tubulin	8547.04	Immunoglobulin C1-set domain	PFAM	1.51E-5
Ferritin	7899.68	Serine-type endopeptidase inhibitor activity	MF	3.27E-5
Myosin	7382.76	Collagen catabolic process	BP	1.09E-4
low complexity protein - collagen-like	6579.48	Homeobox domain	PFAM	1.24E-4
60s ribosomal protein P0	5142.34	CD80-like C2-set immunoglobulin domain	PFAM	1.42E-4
Beta tubulin	4972.09	Metalloendopeptidase activity	MF	1.58E-4
Beta tubulin	4461.74	Cell junction	CC	8.89E-4
Superoxide Dismutase	4220.54	Cadherin domain	PFAM	9.51E-4
Actin	4066.75	Extracellular space	CC	1.77E-3
small heat shock protein 24.1	3933.60	Immunoglobulin V-set domain	PFAM	3.06E-3
Paramyosin	3898.32	Basement membrane	CC	3.57E-3
non coding RNA?	3796.60	Immunoglobulin I-set domain	PFAM	7.51E-3
non coding RNA?	3710.19	Integral component of membrane	CC	7.74E-3
Myosin	3690.69	/	/	/
non coding RNA?	3538.31	/	/	/
Cyclophilin	3464.80	/	/	/
Ribosomal protein S4	3459.95	/	/	/
Adenine nucleotide translocator	3431.98	/	/	/

See the complete list in Additional file 4

*TPM* Transcript Per Million, *PFAM* Pfam conserved domains, *BP* Gene Ontology Biological Process, *MF* Gene Ontology Molecular Function, *CC* Gene Ontology Cellular Component

The most enriched annotations were linked to the extracellular matrix building and remodeling. The included matrixins, Zinc-dependent extracellular proteases involved in the degradation of components of the extracellular matrix [91], metalloendopeptidases and several proteinase inhibitors (e.g. WAP-like proteins, serine-type endopeptidase inhibitors, etc.), which might regulate their enzymatic activity. Consistently with the high prevalence of connective tissue-related genes, annotations linked to cell-cell and cell-extracellular matrix (e.g. immunoglobulin and cadherin domains) were also over-represented. Another class of enriched proteins was that encoded by homeobox genes; while most of these only displayed limited sequence similarity to homeobox genes whose function has been previously characterized, one of these was clearly homologous to Mohawk, a critical regulator of tendon differentiation [92].

### Posterior adductor muscle transcriptional profile

In bivalves, valve closure is controlled by the contraction of the anterior and posterior adductor muscles. However, in all byssus-producing bivalves the anterior muscle is generally much smaller than the posterior one, to the point that in some mussel species (i.e. *Perna* spp.) the anterior muscle scar is not visible anymore. The large posterior adductor muscle is also closely associated to a sinus (the pericardial sac), which is part of the mussel open circulatory system and is commonly used in laboratory practice for the withdrawal of hemolymph with a syringe [93].

The transcriptional profile of the *M. virgata* posterior adductor muscle was particularly poor in terms of transcriptional diversity (TDI = 2.08), being only second to the foot (Fig. 4, panel a). Although the muscle can be certainly considered as a highly specialized tissue, it was characterized by a relatively small number (1089) of tissue-specific genes, probably due to the presence of muscular tissue also in the foot and mantle rim (Fig. 4, panel b; Fig. 5, panel a). As expected, the most highly expressed genes encoded structural components of muscle fibers, including actin and myosin, approximately accounting for 2 and 0.7% of total transcriptional activity, respectively. The enriched annotations contained conserved protein domains commonly associated with muscle adhesion proteins, such as immunoglobulins and fibronectin type III domains, and typical muscle components, such as myofibrils, sarcomere, Z disc, A, I and M bands, and functions, such as telethonin and actinin binding, contraction, response to calcium and sarcomere morphogenesis (Table 7). Curiously, “condensed nuclear chromosome” and “mitotic chromosome condensation” were also listed among the most significantly enriched GO terms. This is linked to the high expression of many contigs corresponding to titin, one of the longest known proteins in metazoans, with over 30,000 amino acids, which mRNA was fragmented in the *Mytilisepta* de novo assembly. Besides its main function as a structural component of the sarcomere, titin has been indeed linked to chromosome stabilization [94].

**Table 7:** The 25 most highly expressed genes in *Septifer virgatus* posterior adductor muscle and representative significantly over-represented annotations by gene set enrichment analysis

Most expressed genes		Over-represented annotations		
Annotation	TPM	Term	Class	*p*-value
Actin	150,779.49	Condensed nuclear chromosome	CC	4.11E-15
60s ribosomal protein L23	69,416.79	Skeletal muscle myosin thick filament assembly	BP	1.09E-14
Actin	44,602.27	Immunoglobulin I-set domain	PFAM	1.89E-14
Paramyosin	39,709.12	Sarcomere organization	BP	1.42E-13
Myosin	28,949.91	M band	CC	2.14E-13
RS-rich protein 1	27,745.06	I band	CC	4.35E-13
Myosin	16,064.48	Structural constituent of muscle	MF	2.31E-12
40S ribosomal protein SA	15,155.63	Muscle alpha-actinin binding	MF	2.64E-12
Myosin	12,525.17	Z disc	CC	3.45E-12
Actin	11,992.63	A band	CC	7.25E-12
Myosin	9275.62	Mitotic chromosome condensation	BP	3.29E-11
Alpha tubulin	8768.82	Myofibril	CC	3.65E-10
Ferritin	8517.40	Myosin filament	CC	5.51E-10
Elongation factor 1 alpha	8411.27	Structural molecule activity conferring elasticity	MF	5.85E-10
PDZ domain containign protein	7804.25	Telethonin binding	MF	5.85E-10
Non coding RNA	5844.46	Sarcomere	CC	5.99E-10
Ribosomal Protein S10	5212.90	Striated muscle thin filament	CC	1.73E-9
Actin	4986.83	Muscle myosin complex	CC	1.73E-9
Non coding RNA	4561.28	Actinin binding	MF	1.73E-9
Alpha tubulin	4344.93	Myosin tail	PFAM	5.07E-7
Unknown	4209.22	Fibronectin type III domain	PFAM	5.33E-6
60s ribosomal protein P0	4157.43	Skeletal muscle thin filament assembly	BP	4.27E-9
Small heat shock protein 24.1	3846.08	Sarcomerogenesis	BP	4.27E-9
Beta tubulin	3725.55	Response to calcium ion	BP	4.86E-7
Superoxide dismutase	3712.29	Muscle contraction	BP	6.84E-7

See the complete list in Additional file 4

*TPM* Transcript Per Million, *PFAM* Pfam conserved domains, *BP* Gene Ontology Biological Process, *MF* Gene Ontology Molecular Function, *CC* Gene Ontology Cellular Component

### Validation of gene expression patterns

**Fig. 6:**
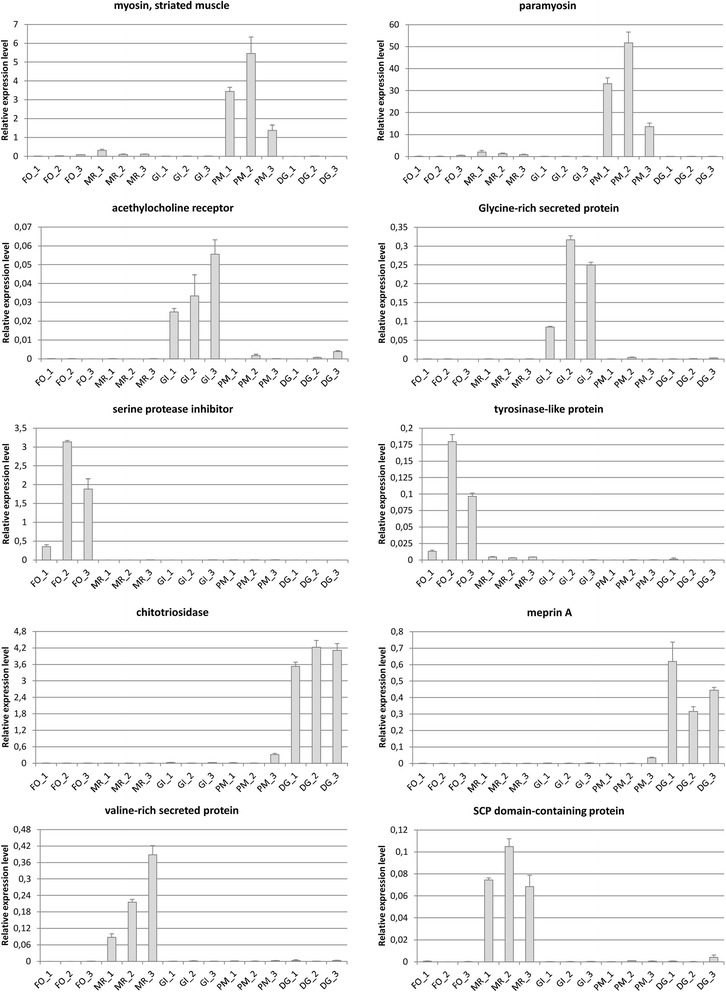
Gene expression levels of 10 selected genes in *M. virgata*. FO: foot; MR: mantle rim; GI: gills; PM: posterior adductor muscle; DG: digestive gland. “1”, “2” and “3” indicate the three biological replicates. *Histogram bars* represent the expression relative to housekeeping genes elongation factor 1 alpha and ribosomal protein S2. Error bars represent the standard deviation of three technical replicates

Taking into account previous reports of high heterozygosity [69] and high inter-individual variability of expression [95] in marine mussels, and considering the fact that RNA sequencing was carried out on a pool of five specimens, we proceeded to a validation of gene expression levels on individual mussels. This was achieved by Real-Time PCR experiments, performed on three biological replicates, which targeted ten tissue-specific genes (Table 1). Overall, the results obtained fully confirmed the observations collected by the in silico analysis of RNA-seq data, supporting our experimental approach for the detection of significant differences of expression among tissues (Fig. 6). In particular, chitotriosidase and meprin A, typical digestive enzymes, displayed high specificity to the digestive gland. Paramyosin and striated muscle myosin, encoding structural components of muscles, were highly expressed only in the posterior adductor muscle. A gills-specific acetylcholine receptor possibly involved in coordinating the movement of cilia and a glycine-rich secreted protein of unknown function were confirmed to be specifically expressed in this multifunctional tissue. A serine protease inhibitor and a tyrosinase-like protein, both important in byssogenesis, were expressed as high levels in the foot, as expected. Finally, an SCP domain-containing protein and a valine-rich protein of unknown function were confirmed as mantle rim-specific gene products.

It is worth noticing that, despite the maintenance of tissue specificity, some of the target genes displayed relevant inter-individual differences in their expression levels. While these variations are not surprising in the light of previous reports [95], they reveal that a number of unpredictable factors of difficult monitoring might influence gene expression of *M. virgata* in the marine environment. As an example, the two foot-specific genes displayed an expression level more than 10 times lower in specimen 1 compared to the two other biological replicates (Fig. 6). This difference might indicate a lower byssogenic activity, a process which is well known to be influenced by multiple environmental parameters [96], including the intensity of water currents and exposure to waves, factors which could not be controlled in the sites of sampling.

In summary, the results obtained by RT-PCR confirmed that the pooling approach we used was appropriate to detect major differences in gene expression among tissues, permitting to pinpoint gene products which cover an important physiological function in the different body districts of mussels. At the same time however, the non-negligible inter-individual differences observed point out that extra care should be taken into account in future experiments aimed at assessing the individual response of *M. virgata* to abiotic and biotic stress.

### The high prevalence of lectins in the *Mytilisepta virgata* transcriptome

Bivalve mollusks are known to produce a very broad range of secreted proteins containing carbohydrate-binding domains (CRDs). It has been suggested that these lectin-like molecules might function as Pattern Recognition Receptors (PRRs), acting as a first line of defense towards pathogens. Indeed, due to their high sequence diversity, bivalve lectins could potentially broaden the spectrum of recognition for Microbe Associated Molecular Patterns (MAMPs) or PAMPs, depending on whether the whole microbiota or just its pathogenic fraction are taken into account. However, alternative functions have been suggested. Specifically, the lectins found in the mucus that covers digestive organs have been linked to the feeding process, thanks to their ability to recognize carbohydrates exposed on the surface of food particles [97]. Others might be involved in establishing genetic barriers between species by regulating the species-specific recognition of congenetic gametes [98–100].

Many different types of bivalve lectins have been isolated since the early ‘90 through classical biochemical methods [101–103] and many more have been discovered in recent years in different bivalve species. Although most of these pertain to four distinct families, i.e. C-type lectins, fibrinogen-related proteins (FREPs), galectins and C1qDC proteins, others have been also found, including SUEL-type lectins, [104], lipopolysaccharide and β-1,3-glucan binding proteins [105], mytilectins [106] and F-type lectins [107].

Besides covering fundamental roles in bivalve physiology, some bivalve lectins also possess highly interesting binding or cytotoxic properties which might warrant future studies aimed at developing potential biotechnological applications. For example, the recently identified MytiLec is cytotoxic against some cancer cell types [108], and it has been suggested that a hemolytic C-type lectin from the freshwater clam *Villorita cyprinoides* could be potentially used as a clot lysing molecule [109].

Some bivalve lectin-encoding gene families are extremely expanded and comprise several hundred members, as suggested by proteomic [110] and transcriptome studies [20, 111, 112] and later confirmed on a whole genome scale in oyster [113]. The purplish Japanese mussel is no exception, as some important lectin-like gene families were also found among the most abundant ones in the de novo assembled transcriptome (Table 8).

**Table 8:** The 25 most highly represented PFAM conserved domains in the *Mytilisepta virgata *transcriptome

Domain name	PFAM code	domain count
Protein kinase domain	PF00069	200
Immunoglobulin domain	PF00047	190
Protein tyrosine kinase	PF07714	183
Ankyrin repeats (3 copies)	PF12796	182
Ankyrin repeats (many copies)	PF13857	175
EF-hand domain pair	PF13833	161
**C1q domain**	PF00386	161
Immunoglobulin I-set domain	PF07679	161
Ankyrin repeat	PF00023	160
EF hand	PF00036	157
**Lectin C-type domain**	PF00059	154
EGF-like domain	PF00008	149
EF-hand domain	PF13405	147
AAA domain	PF00004	139
Zinc finger, C3HC4 type (RING finger)	PF00097	129
50S ribosome-binding GTPase	PF01926	118
Leucine rich repeat	PF13855	117
7 transmembrane receptor (rhodopsin family)	PF00001	117
WD domain, G-beta repeat	PF00400	110
Immunoglobulin V-set domain	PF07686	109
B-box zinc finger	PF00643	108
von Willebrand factor type A domain	PF00092	106
Leucine Rich repeats (2 copies)	PF12799	103
Tetratricopeptide repeat	PF00515	103
Zinc finger, C2H2 type	PF00096	102
**F5/8 type C domain**	PF00754	42
**Fibrinogen C-terminal globular domain**	PF00147	37
**Galactose binding lectin domain**	PF02140	23
**Ricin-type beta-trefoil lectin domain-like**	PF14200	6
**H-type lectin domain**	PF09458	6
**Galactoside-binding lectin**	PF00337	2

Domains with carbohydrate-binding properties are marked in bold. A few additional less abundant lectin-like domains are listed in the bottom part of the table

**Fig. 7:**
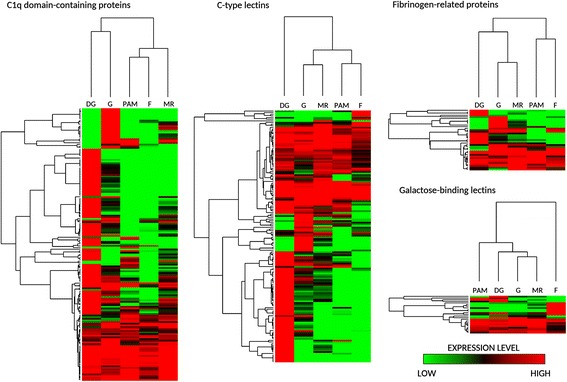
Heat maps summarizing gene expression patterns of C1q domain-containing proteins, C-type lectins, Fibrinogen-related proteins and Galactose-binding lectins in *Mytilisepta virgata*. Heat maps were built with log_2_ transformed gene expression levels; genes and tissues were hierarchically clusterized based on Euclidean distances, estimated with average linkage method. DG: digestive gland; G: gills; PAM: posterior adductor muscle; F: foot; MR: mantle rim

Similarly to other mussel species [38], C1qDC proteins were found in very high number in *M. virgata* (161), suggesting the presence of several hundred genes in the genome of this species, in line with the previously demonstrated massive lineage-specific C1q gene family expansion event which occurred in the class Bivalvia [87]. C1qDC proteins have been implicated on multiple occasions in the innate immune response of different mollusks, where they have been often designed as sialic acid-binding lectins [114–116]. The expression profile of C1qDC genes in *Mytilisepta* closely resembles that of the Pacific oyster [87], since the most of such genes are selectively expressed in the digestive gland or in the gills. However, ~25% of mussel C1qDC proteins are produced ubiquitously by all tissues (Fig. 7).

C-type lectins represent another large family of carbohydrate-binding proteins, comprising 154 members in *M. virgata*. These proteins, which structurally resemble the mannose-binding lectin (MBL) involved in the lectin pathway of the vertebrate complement system, have been often linked to bivalve immunity as PRRs [117–119]. However, coherently with their remarkable structural diversification, they could also be involved in other functions, such as particle capture [120] and gamete recognition [98]. Overall, the pattern of expression of C-type lectins was similar to that of C1qDC proteins, with two well-distinct groups of digestive gland- and gills- specific genes and others found at similar levels in all tissues (Fig. 7).

The role of FREPs, first described in gastropod mollusks [88], has later been demonstrated also in bivalves [121, 122]. Despite inconclusive phylogeny, molluscan FREPs structurally resemble vertebrate ficolins which, like MBL, can activate the complement system through the lectin pathway. FREPs possess an astounding sequence diversity in *Mytilus* spp., where they are though to provide a broad array of PRRs [123]. A total of 37 different FREPs could be identified in the purplish mussel, where they appear to be expressed in a broad range of tissues, but in particular in the gills (Fig. 7).

A remarkable number of proteins bearing a galactose-binding domain (23) was also found to be encoded by *Mytilisepta* mRNAs. This domain, first identified in SUEL lectins from sea urchin eggs, allows the preferential binding of L-rhamnose, in addition to D-galactose [124]. Although most of them are expressed in all the main tissues of mussels, some are clearly foot-specific. On the other hand, the galactoside-binding domain, which characterizes galectins, was only identified in two sequences encoding two structurally different galectins with two and four consecutive CRDs, respectively.

Consistently with previous reports in *M. galloprovincialis* [20], the F5/8 type C domain, typical of fucose-binding (F-type) lectins, was also detected in high abundance (42) in the *Mytilisepta* transcriptome. However, the function of F-type lectins has been only marginally investigated in bivalves so far and, taking into account that the F5/8 type C domain is also found in many non-lectin proteins (e.g. coagulation factors and bindins), the data currently available is insufficient to classify the *M. virgata* sequences as bona fide F-type lectins.

Six different proteins bearing a ricin-type beta-trefoil lectin-like domain are present in the de novo assembled transcriptome. However, none of these can be classified as mytilectins, despite the fact these novel carbohydrate binding proteins have been first characterized and described as a multigenic family in *M. galloprovincialis* [106, 108]. This unexpected absence may either reflect a lack of expression in physiological conditions or a highly specific expression to one of the tissues which could not be sampled in the current experiment (e.g. hemocytes or inner mantle).

The H-type lectin domain is capable of binding N-acetylgalactosamine and it has been described in the agglutinin of land snails, proteins which are part of the innate immune system, protecting fertilized eggs from bacteria [125, 126]. Although to date no H-type lectin has ever been characterized in bivalve mollusks, six assembled transcripts pertain to this family in *M. virgata*.

Altogether these data confirm the high prevalence of transcripts encoding lectins in bivalve transcriptomes. Although the large majority of the putative carbohydrate-binding proteins encoded by the transcripts identified in *M. virgata* either pertaining to the C1qDC or to the C-type lectin families, many others, with distinct binding properties and potential biotechnological applications, are also present. Overall, gene expression data point out the digestive gland as the most lectin-rich tissue, followed by the gills, while on the other hand the mantle rim, foot and posterior adductor muscle only produce a limited set of lectin-like molecules. While this remarkable expression might be linked to food particle selection, it is also consistent with the emerging role of peripheral tissues in the bivalve innate immune system, coherently with the extensive contact of gills and digestive tract with the external environments and microbes associated with sea water, sediment and food particles.

## Conclusions

As more and more bivalve species become the subject of –omic studies thanks to the development of cost-effective sequencing technologies, most transcriptomic studies are usually either focused on single tissues or on samples obtained from whole body, and just a very few have been so far dedicated to the investigation of the highly specialized function of tissues in a comparative way. With the present study, we tried to fill a gap in the genetic knowledge of *M. virgata*, providing a detailed snapshot of the gene expression profile of most of the main tissues of this marine mussel species. Besides revealing the high tissue-specificity of genes fundamental for mussel immune defense, feeding and attachment to the substrate, we also provide compelling evidence bimolecular phylogeny that the Japanese purplish mussel is not closely related to *Septifer* (Récluz, 1848) and that is should be instead considered as part of the Brachidontinae subfamily.

## Additional files

Additional file 1: Annotations and gene expression levels of *Mytilisepta virgata* contigs. (XLSX 5900 kb)

Additional file 2: Multiple sequence alignment of the consensus of the 17-mer repeated units present in the byssal cuticle proteins of *Mytilisepta virgata*, *Septifer biforcatus* and *Bathymodiolus thermophilus*. (PDF 122 kb)

Additional file 3: Scatter plots representing paired comparisons of gene expression profiles between tissues. (PDF 1316 kb)

Additional file 4: Complete results of gene set enrichment analyses. (XLSX 29 kb)

## Abbreviations

BUSCO: Benchmarking Universal Single-Copy Ortholog
C1qDC: C1q domain-containing
CDS: Coding sequence
COI: Cytochrome c oxidase subunit I
CRD: Carbohydrate recognition domain
DEG: Differentially expressed gene
FREP: Fibrinogen-related protein
L-DOPA: L-3,4-dihydroxyphenylalanine
MAPM: Microbe Associated Molecular Pattern
PAMP: Pathogen Associated Molecular Pattern
PCR: Polymerase chain reaction
PRR: Pattern Recognition Receptor
TDI: Transcriptomic Diversity Index
TPM: Transcripts Per Million
