# Supplementary material for: The purplish bifurcate mussel Mytilisepta virgata gene expression atlas reveals a remarkable tissue functional specialization
Source: BMC Genomics. 2017 Aug 8;18:590. doi: 10.1186/s12864-017-4012-z (PMC5549309; doi:10.1186/s12864-017-4012-z)

Supplementary File 2

Multiple sequence alignment of the consensus of the 17-mer repeated units present in the byssal cuticle proteins of *Mytilisepta virgata*, *Septifer biforcatus* and *Bathymodiolus thermophilus*. Sequence logo of the consensus is shown in detail for *B. thermophilus* and *M. virgata*.

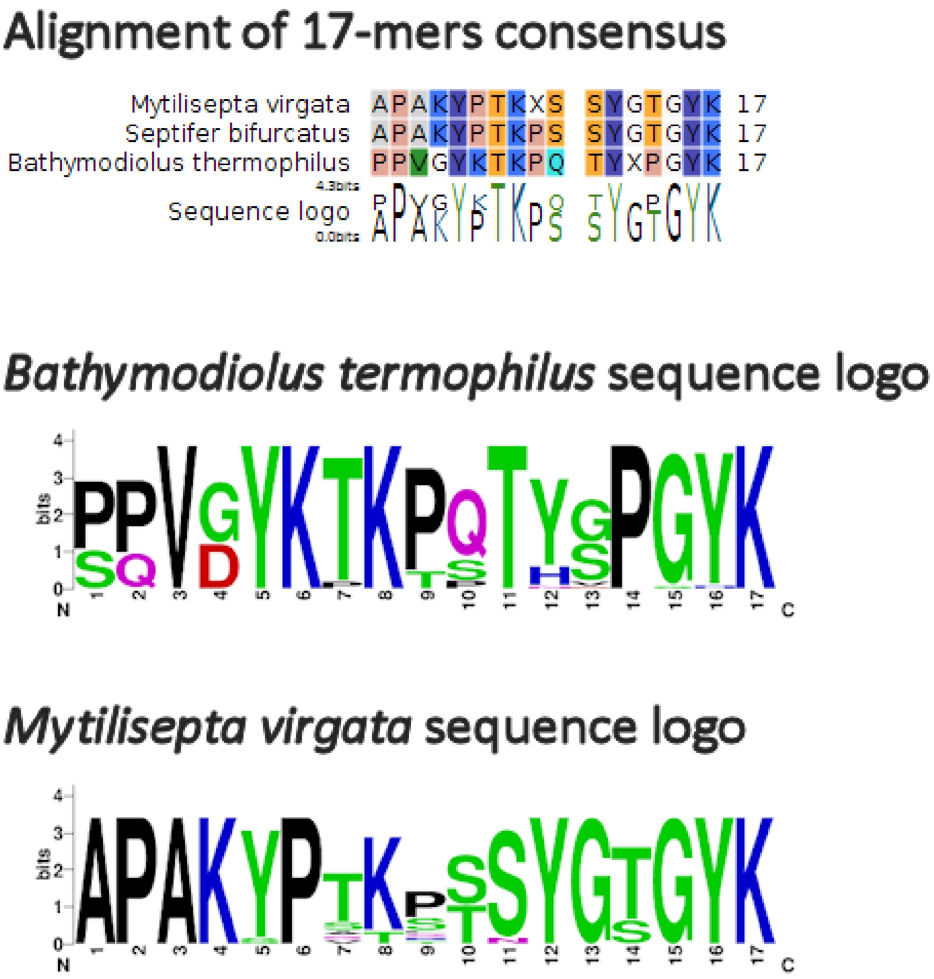

Supplement: Supplementary file 2 — Multiple sequence alignment of the consensus of the 17-mer repeated units present in the byssal cuticle proteins of Mytilisepta virgata, Septifer biforcatus and Bathymodiolus thermophilus. (PDF 122 kb) [file 12864_2017_4012_MOESM2_ESM.pdf]
