# Supplementary material for: The purplish bifurcate mussel Mytilisepta virgata gene expression atlas reveals a remarkable tissue functional specialization
Source: BMC Genomics. 2017 Aug 8;18:590. doi: 10.1186/s12864-017-4012-z (PMC5549309; doi:10.1186/s12864-017-4012-z)

DIGESTIVE GLAND vs FOOT

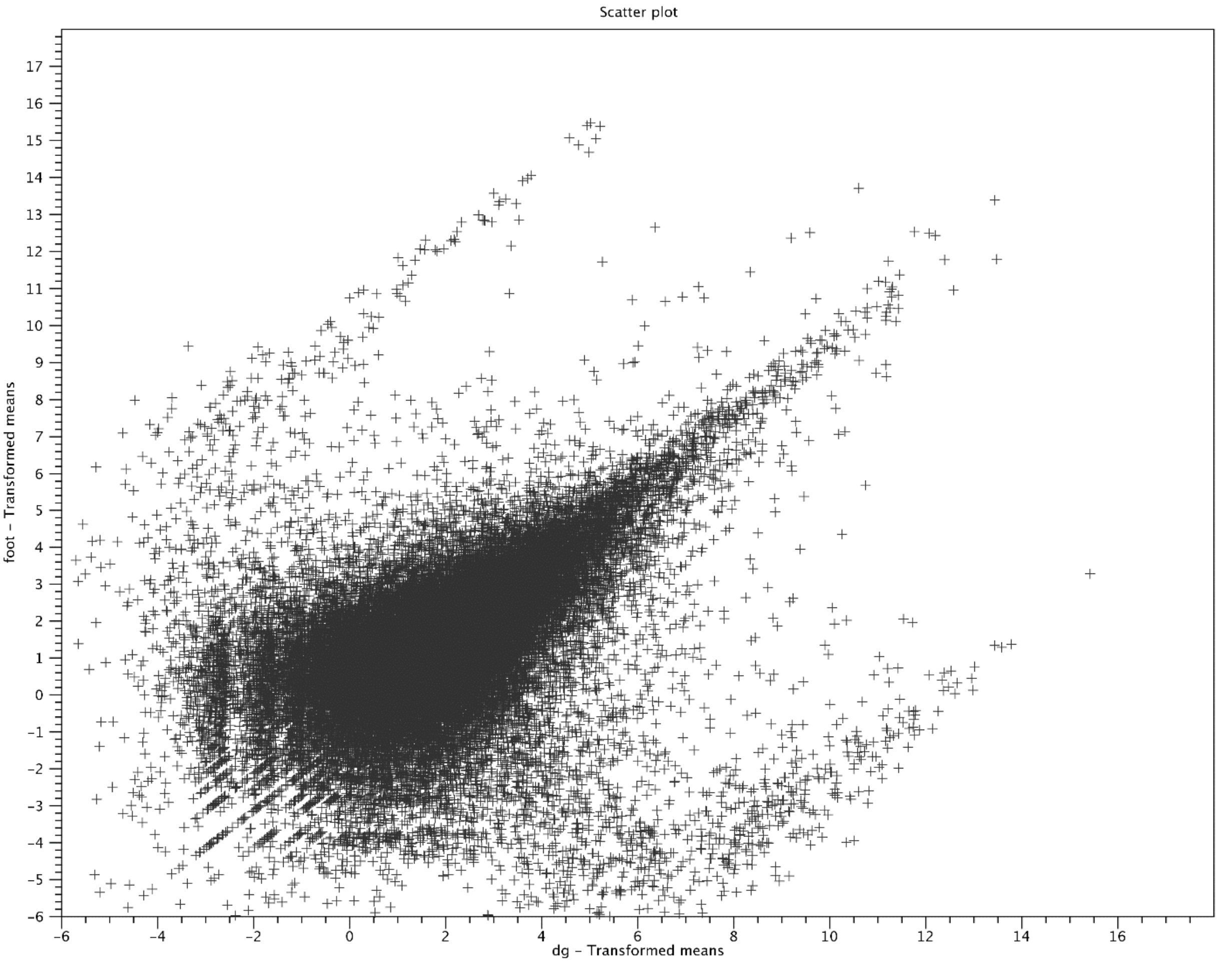

DIGESTIVE GLAND VS GILLS

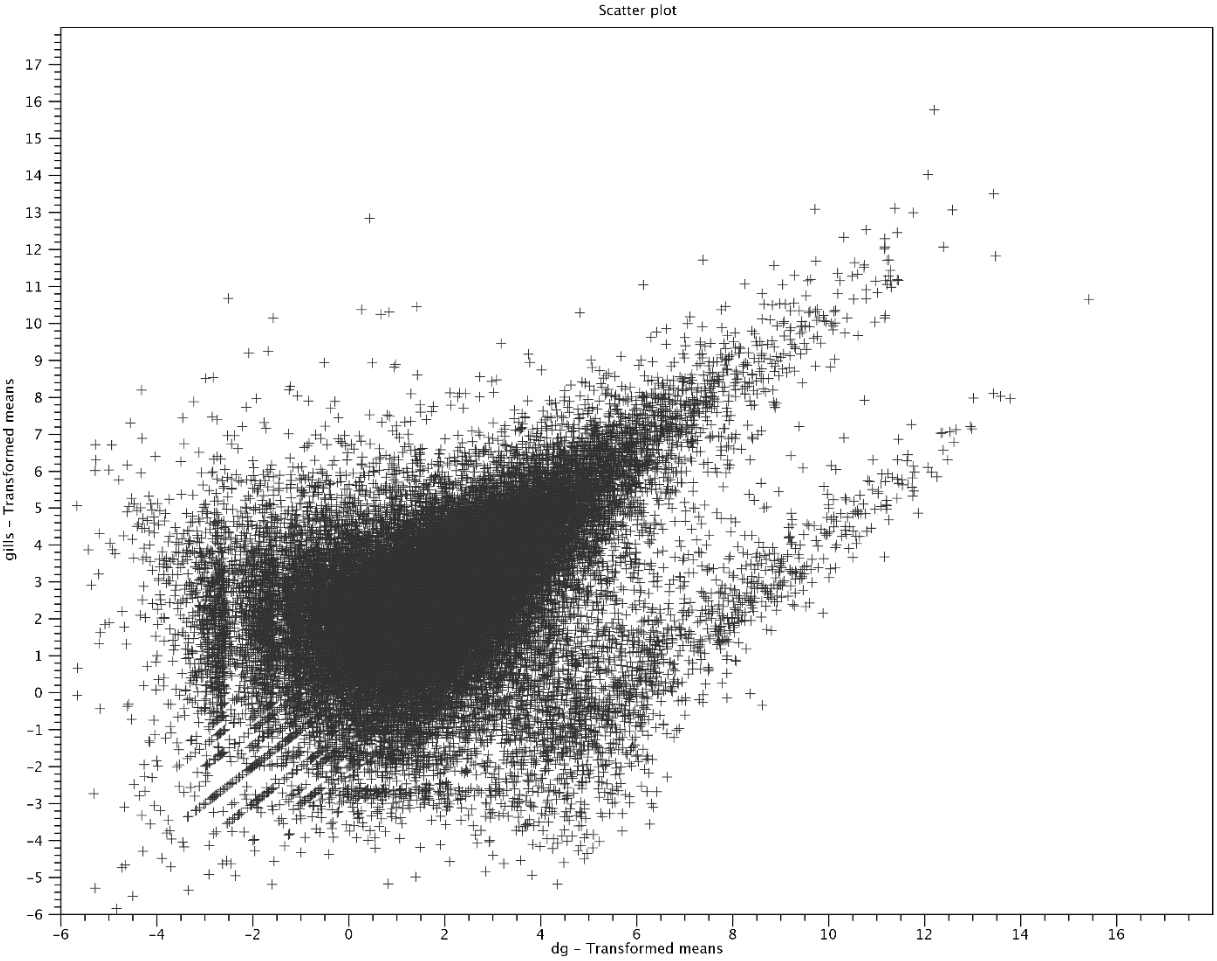

DIGESTIVE GLAND vs MANTLE RIM

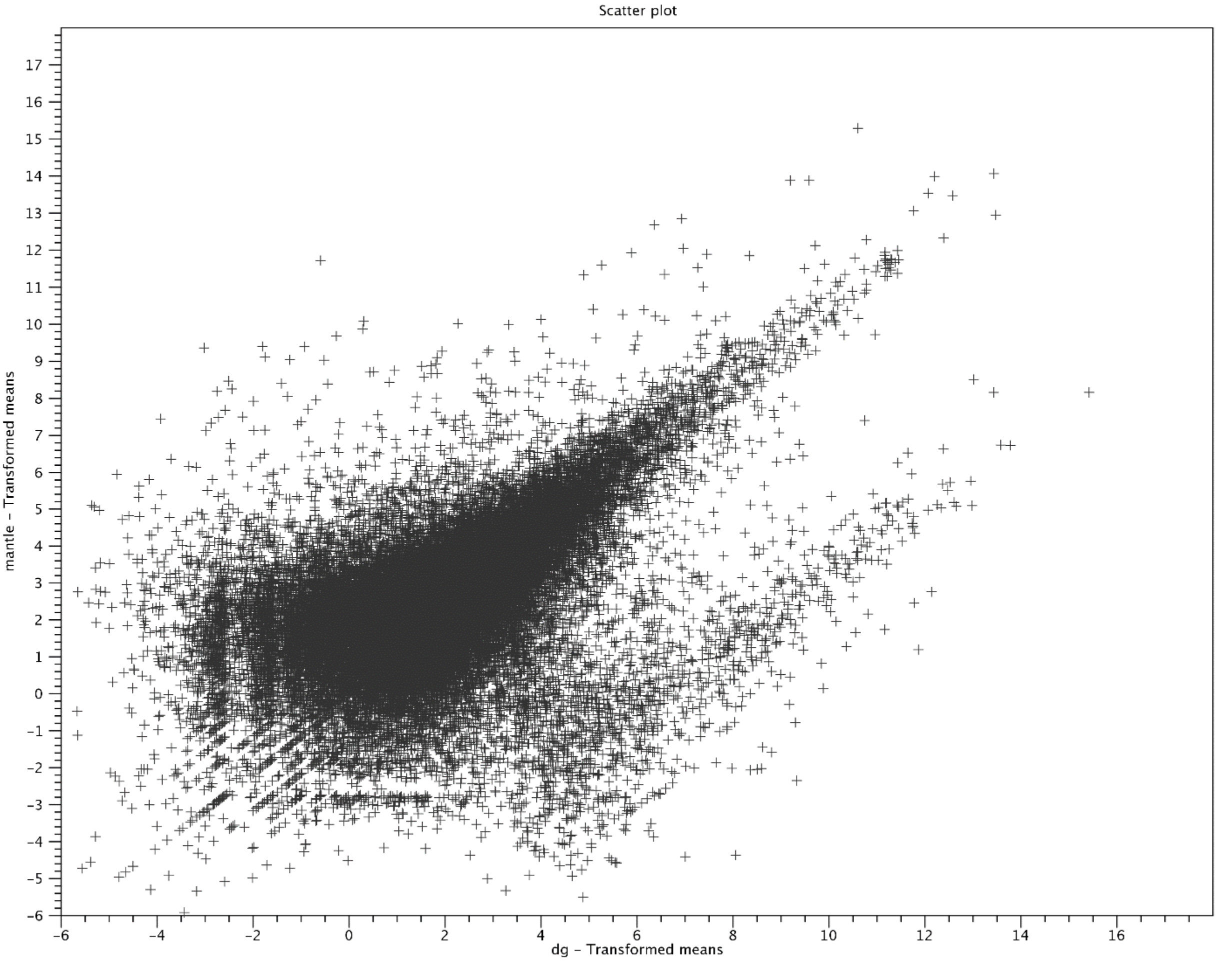

DIGESTIVE GLAND VS POSTERIOR ADDUCTOR MUSCLE

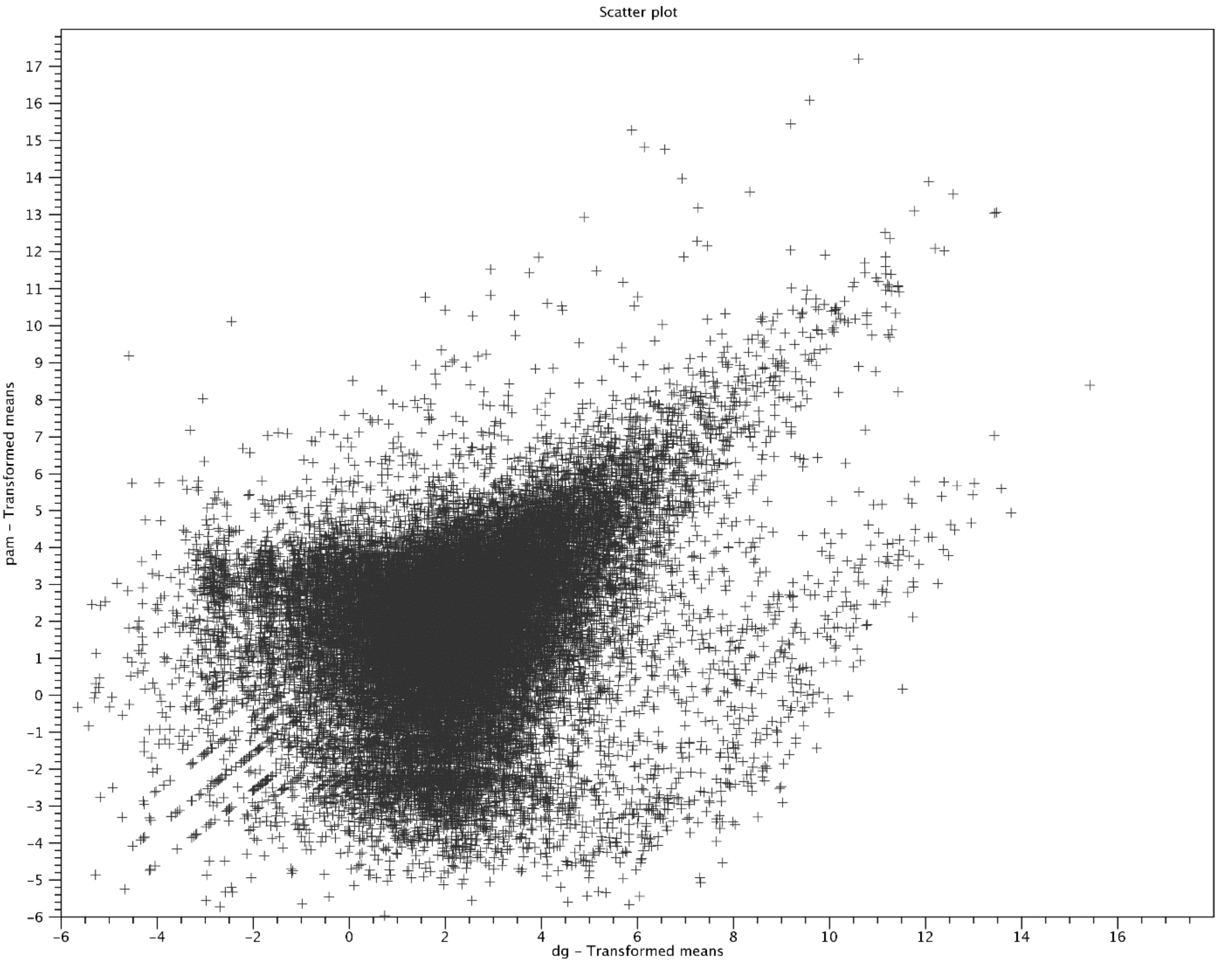

GILLS VS FOOT

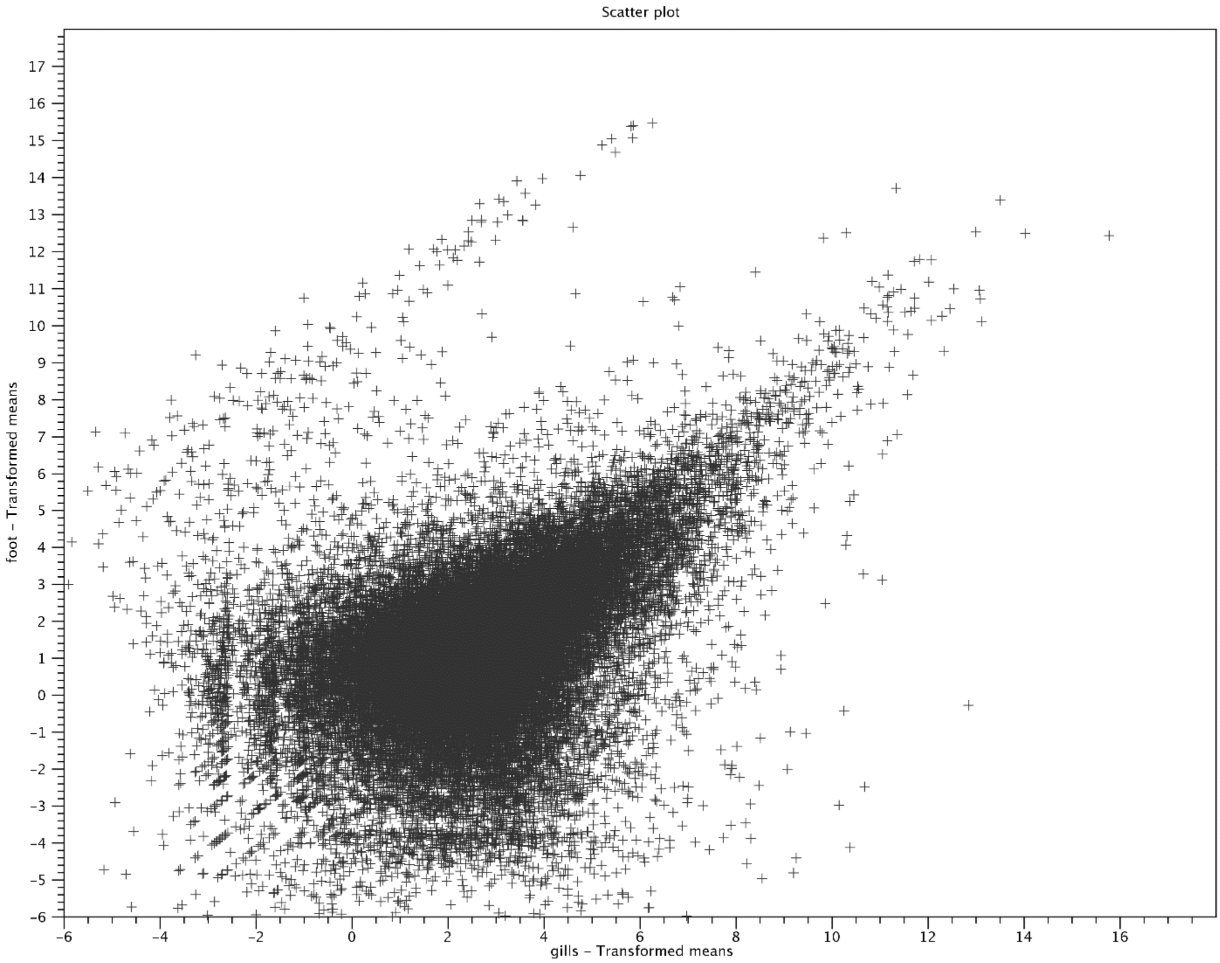

GILLS VS MANTLE RIM

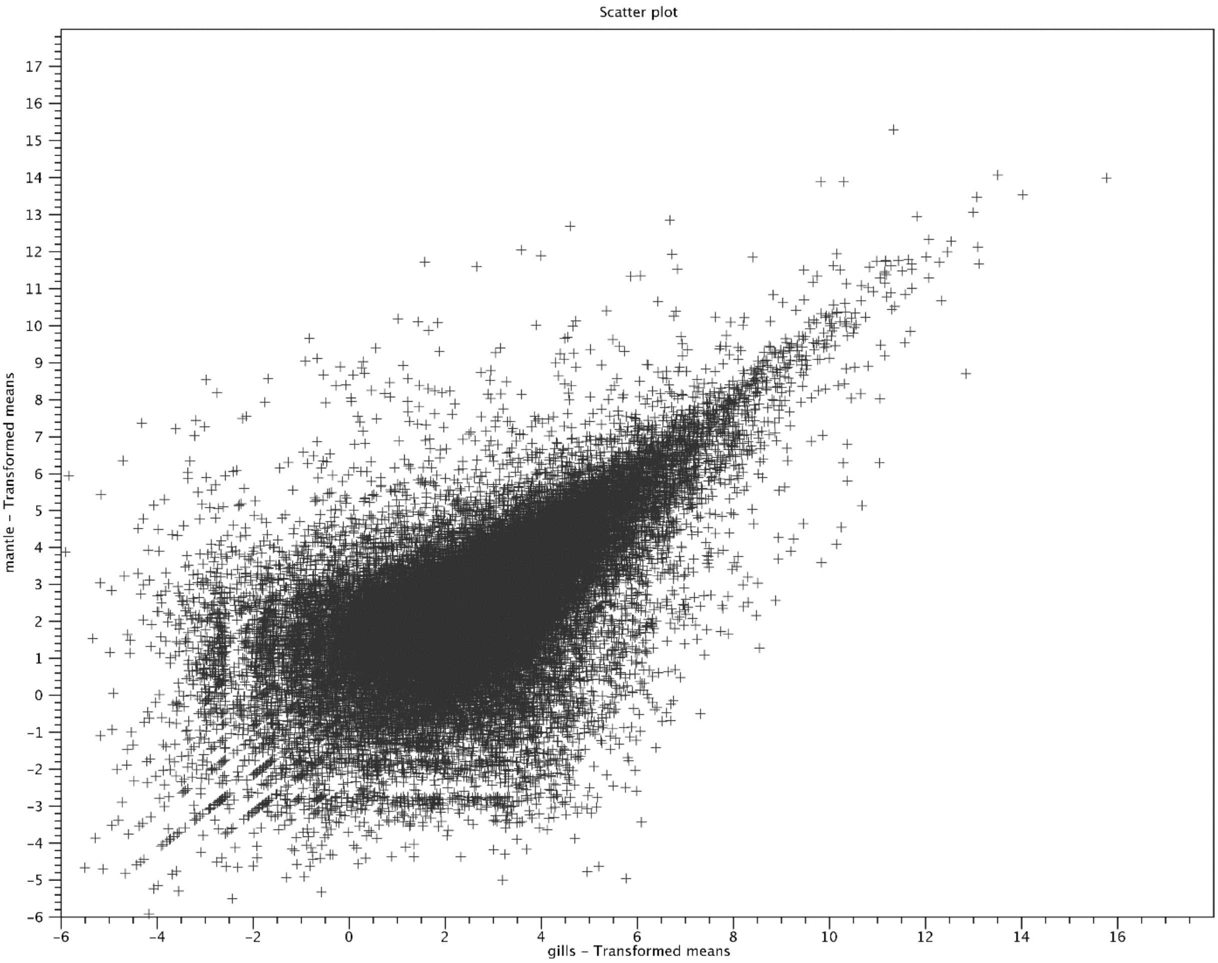

MANTLE RIM vs FOOT

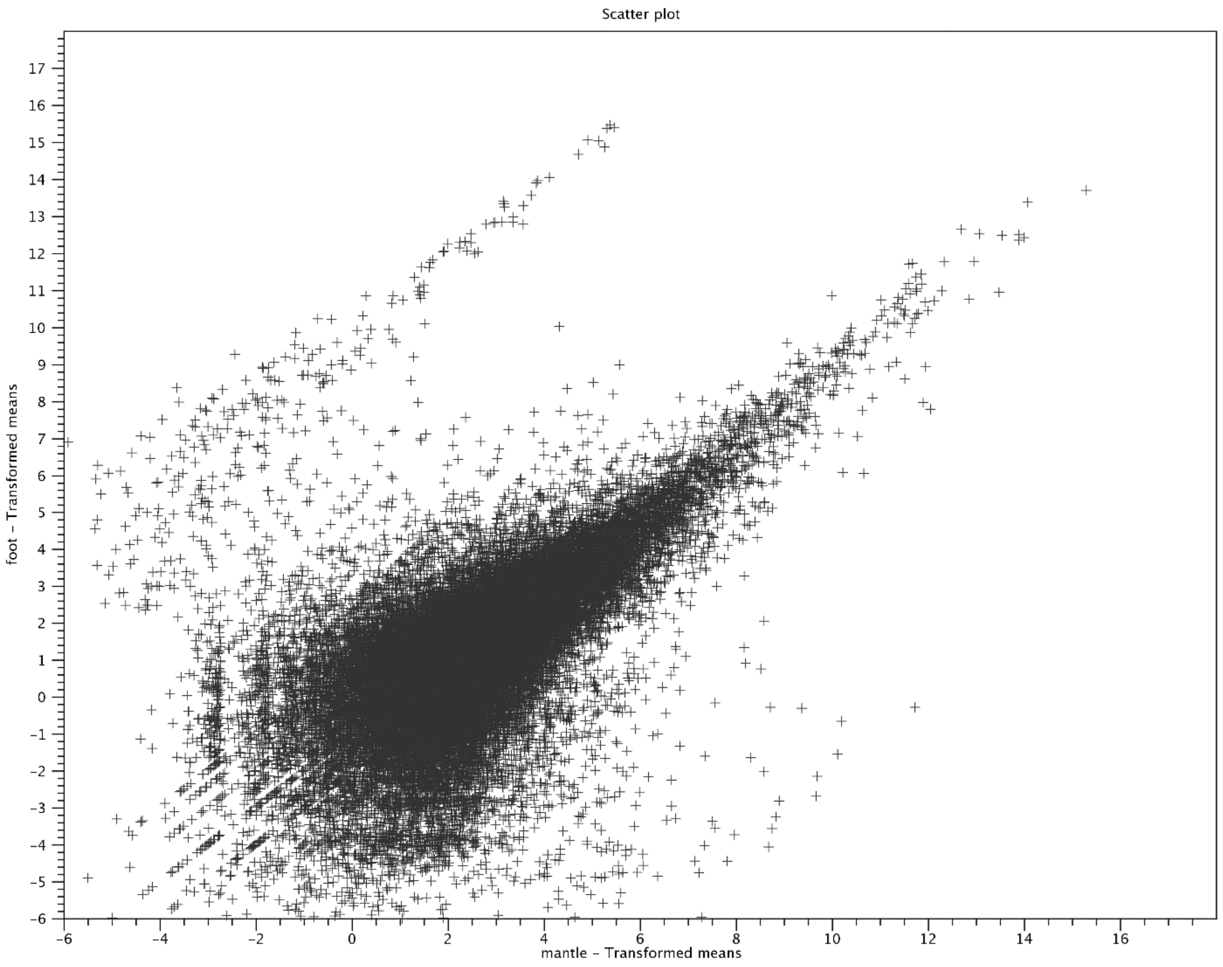

POSTERIOR ADDUCTOR MUSCLE VS FOOT

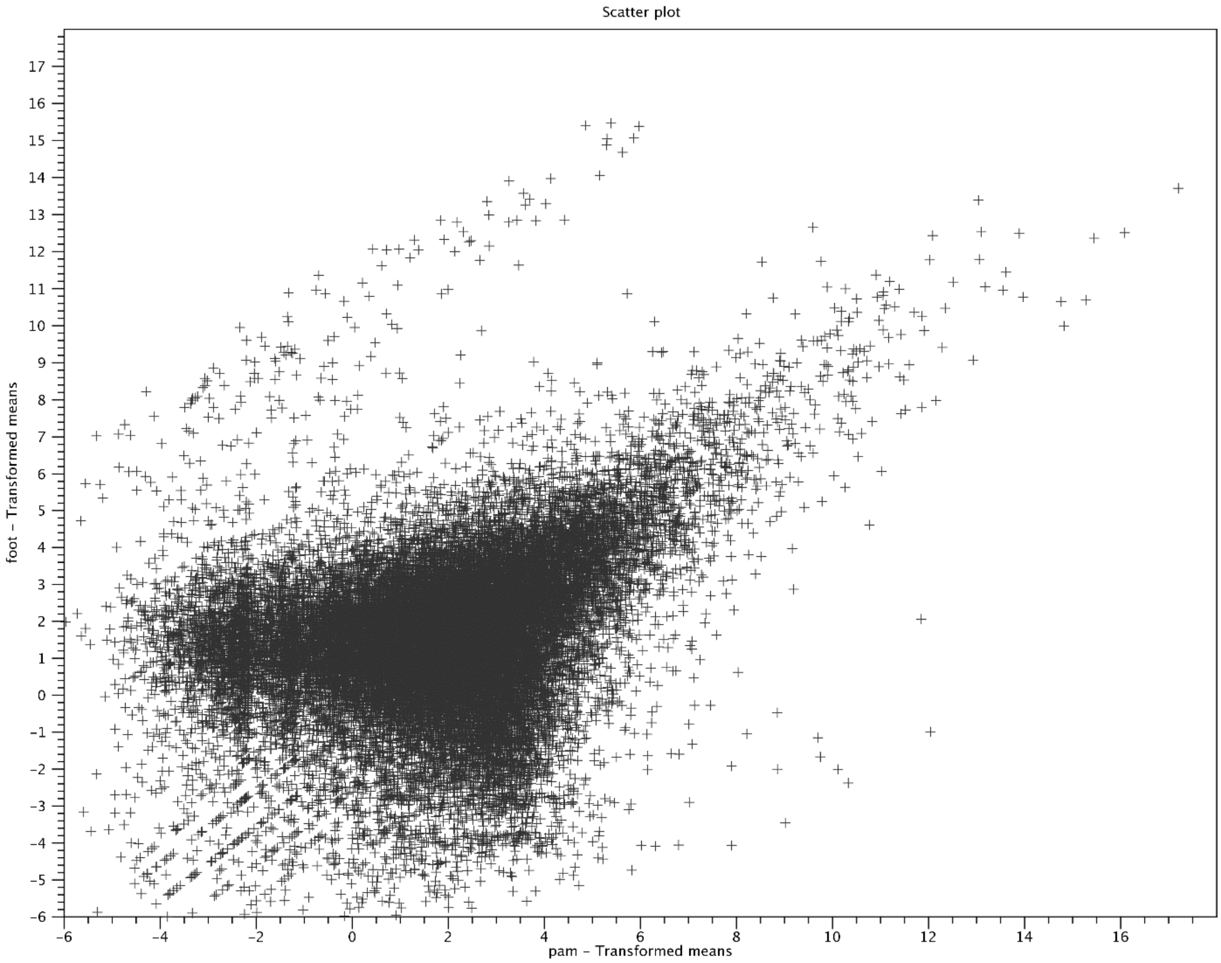

POSTERIOR ADDUCTOR MUSCLE VS GILLS

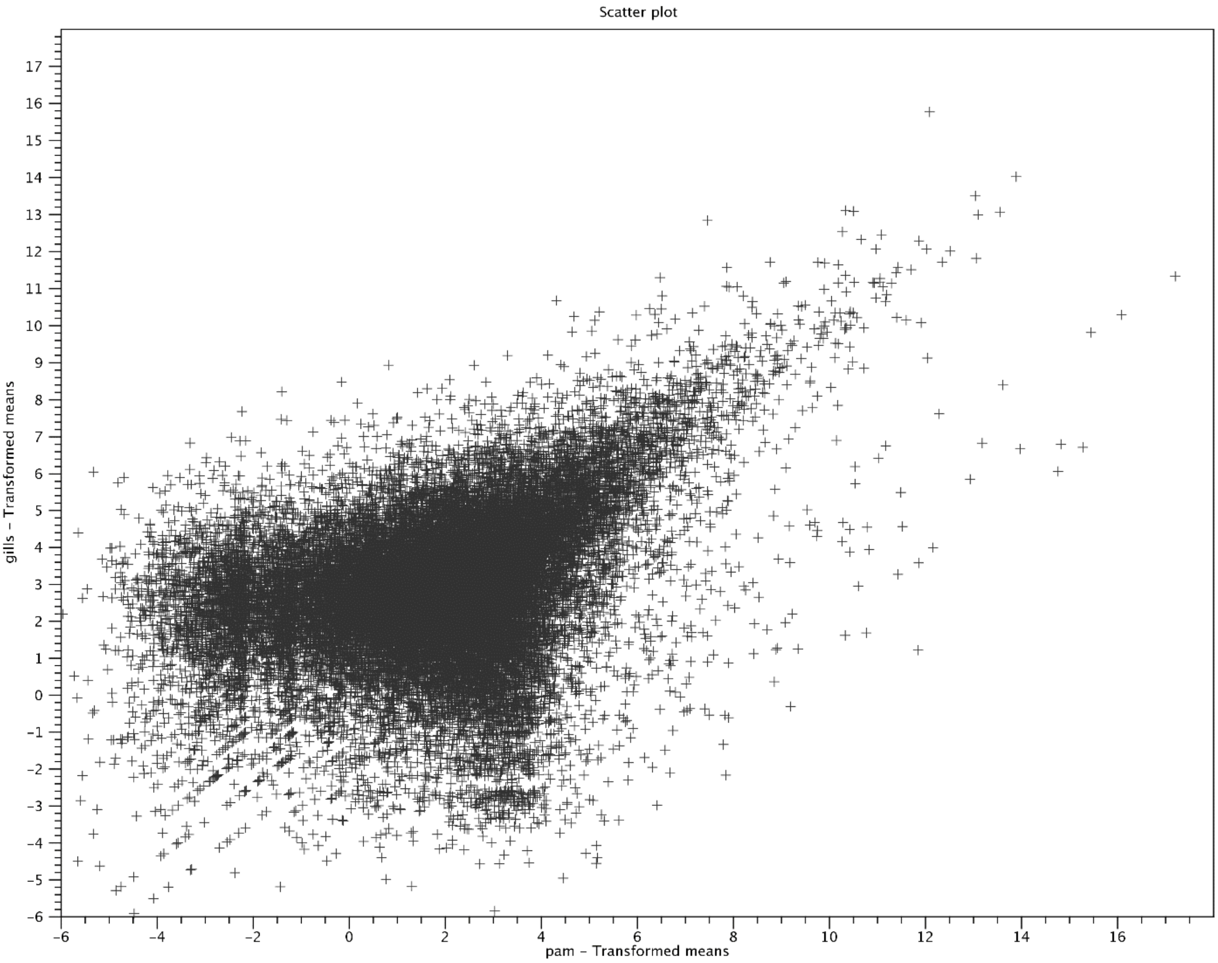

POSTERIOR ADDUCTOR MUSCLE VS MANTLE RIM

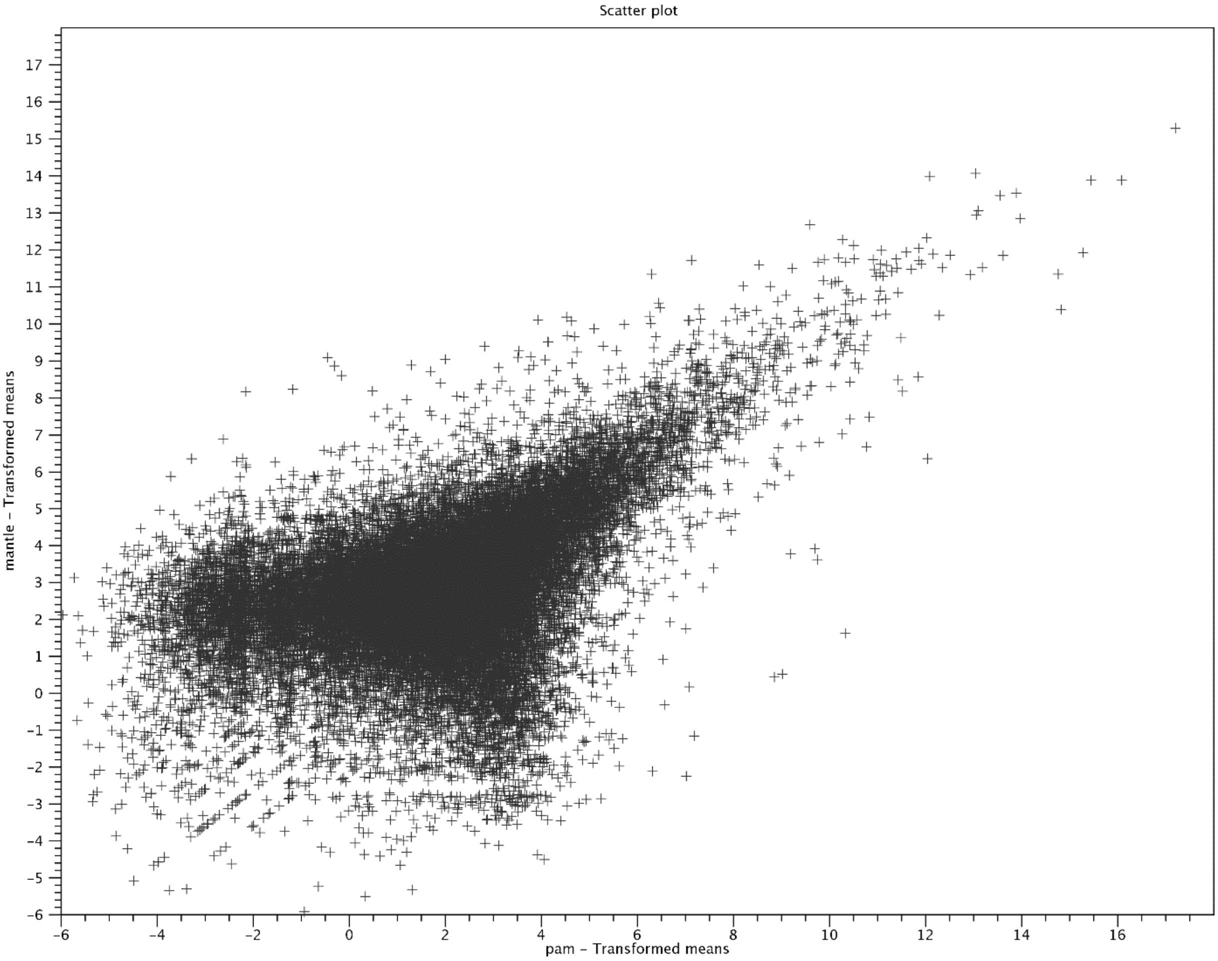

Supplement: Supplementary file 3 — Scatter plots representing paired comparisons of gene expression profiles between tissues. (PDF 1316 kb) [file 12864_2017_4012_MOESM3_ESM.pdf]
